# Chili-Seg: a high-precision detection and instance segmentation model for chili flowers in complex environments

**DOI:** 10.3389/fpls.2026.1955273

**Published:** 2026-09-29

**Authors:** Minqiu Kuang, Xiaojian Li, Zhizhen Hu, Dawei Liu, Yang Xiang, Fangping Xie, Yuxuan Zhang, Xu Li

**Affiliations:** 1 College of Electrical and Mechanical Engineering, Hunan Agricultural University, Changsha, China; 2 Foshan-Zhongke Innovation Research Institute of Intelligent Agriculture and Robotics, Foshan, China; 3 Hunan Provincial Key Laboratory of Intelligent Agricultural Machinery Equipment, Changsha, China; 4 College of Intelligent Science and Engineering, Beijing University of Agriculture, Beijing, China; 5 Department of Computer and Electrical Engineering, Mid Sweden University, Sundsvall, Sweden

**Keywords:** breeding, chili flower, complex environments, deep learning, instance segmentation

## Abstract

Accurate recognition and instance segmentation of chili flowers are fundamental to the development of intelligent breeding robots and the improvement of operational efficiency in protected agriculture. However, in unstructured field environments, chili flowers are typically small-scale targets and are frequently affected by occlusion from leaves and branches as well as illumination variations, leading to degraded feature representation and suboptimal segmentation performance in existing models. To address these challenges, this study proposes a high-precision detection and segmentation framework, termed Chili-Seg, built upon the YOLO11n-seg baseline architecture (YOLO: You Only Look Once). To enhance detection and segmentation performance, an Efficient Head is introduced to replace the original decoupled head. By optimizing the parameter-sharing mechanism between classification and regression branches, the proposed head improves both localization accuracy and segmentation quality while maintaining computational efficiency. To further strengthen feature extraction under complex field conditions, a C2f with Dynamic Position Bias (C2DPB) module is designed to replace the original C2PSA module based on Position-Sensitive Attention (PSA) in the backbone network. The dynamic position-bias mechanism enhances the representation of fine-grained morphological characteristics of chili flowers while effectively suppressing background interference, thereby reducing mis-segmentation caused by environmental noise. Moreover, a Generalized Dynamic Feature Pyramid Network (GDFPN) is introduced to reconstruct the neck architecture. By enabling dynamic cross-scale feature interaction and adaptive weighted fusion, the proposed design mitigates feature loss caused by large variations in object scale and significantly improves multiscale feature representation capability. Experimental results on a self-constructed chili flower dataset demonstrate that Chili-Seg achieves improved overall instance-segmentation performance compared with representative YOLO-based baselines, including YOLOv5n-seg and YOLO11nseg; semantic-segmentation models are evaluated separately using their corresponding pixel-level metrics. For detection tasks, the proposed method achieves a precision of 96.8%, mean Average Precision at an Intersection over Union (IoU) threshold of 0.50 (*mAP*_50_) of 90.6%, and *mAP*_50–95_, averaged over IoU thresholds from 0.50 to 0.95, of 77.9%, improving the baseline by 1.7, 1.2, and 1.8 percentage points, respectively. For segmentation tasks, it achieves a precision of 96.6%, *mAP*_50_ of 89.8%, and *mAP*_50–95_ of 64.7%, with improvements of 2.1, 1.1, and 1.3 percentage points over the original model. Furthermore, the proposed model is deployed on the NVIDIA Jetson AGX Orin platform for real-time evaluation, achieving a processing speed of 102.4 frames per second (FPS). The results indicate that Chili-Seg achieves a favorable trade-off between accuracy, segmentation quality, and real-time performance, providing a robust foundation for subsequent extraction of chili flower phenotypic parameters.

## 1 Introduction

### 1.1 Introduction

Crop phenotypes serve as an important bridge linking plant genotypes with environmental responses and provide critical information for precision breeding, crop monitoring, and high-throughput selection (Yang et al., 2020; Araus and Cairns, 2014; Jin et al., 2022; Kuang et al., 2026j, g). With the rapid development of imaging sensors and high-throughput phenotyping platforms, automated extraction of phenotypic traits from plant organs has become a major research focus in smart agriculture and digital breeding (Li et al., 2014; Paulus, 2019; Kuang et al., 2026c). Chili pepper (*Capsicum annuum* L.) is an economically important horticultural crop, and reliable perception of floral organs is relevant to flowering monitoring, breeding operations, and robotic pollination (Kuang et al., 2026d, h). Accurate identification and instance segmentation of chili flowers therefore provide important visual information for quantitative flower phenotyping and intelligent pollination systems (Kuang et al., 2025, 2026b, f). Unlike bounding-box detection, instance segmentation can distinguish adjacent or overlapping flower instances and provide pixel-level contours, which are required for morphology measurement and for extracting spatial cues used by downstream robotic operations.

Despite recent progress in agricultural computer vision, reliable perception of chili flowers under field conditions remains challenging. Compared with fruits and mature organs, chili flowers are typical small-scale targets characterized by weak texture, irregular morphology, and limited pixel occupancy. In practical cultivation environments, dense foliage, severe occlusion, illumination variation, and complex background interference often result in blurred boundaries and incomplete feature representation (Zhang et al., 2026b, c, d). Deep learning-based object detection frameworks have significantly advanced agricultural vision applications, particularly the You Only Look Once (YOLO) family, which provides a favorable balance between accuracy and inference speed and has been applied to fruit, flower, and crop monitoring tasks (Mirhaji et al., 2021; Wang et al., 2022b; Moreira et al., 2025; Lu et al., 2026; Zhang et al., 2026a; Dong et al., 2025; Kuang et al., 2026h, a, i, e). Meanwhile, Mask Region-based Convolutional Neural Network (Mask R-CNN) introduced instance-level mask prediction and has been successfully applied to agricultural organ segmentation under complex backgrounds (Wang and He, 2022; Tian et al., 2020; Yu et al., 2019). More recently, Transformer-based architectures have advanced global contextual modeling. Representative models such as Vision Transformer (ViT), Swin Transformer, Mask2Former, and OneFormer have achieved strong performance across image recognition and segmentation benchmarks (Dosovitskiy et al., 2021; Liu et al., 2021; Cheng et al., 2022; Jain et al., 2023). However, these architectures can require substantial computational resources and memory, which can complicate deployment on resourceconstrained agricultural robotic platforms. Furthermore, lightweight segmentation networks still face challenges in preserving fine-grained boundary information and small-object semantics during repeated downsampling and multi-scale feature fusion.

To address these compounding challenges, this study introduces Chili-Seg, a novel phenotype detection and instance segmentation model tailored for unstructured environments, developed using YOLO11n-seg as the baseline framework. By systematically engineering the detection head architecture, backbone feature extraction mechanism, and multi-scale feature fusion network, the proposed model significantly enhances perceptual sensitivity toward complex field backgrounds and multi-scale target variations. The primary contributions of this work are summarized as follows:

- Development of the Chili-Seg Framework: Tailored for unstructured field environments, we propose Chili-Seg, a lightweight yet highly accurate instance segmentation framework specifically engineered for the robust detection and boundary delineation of chili flowers.
- Architectural Engineering of the Efficient Head: To maximize localization and segmentation precision, we introduce an Efficient Head that replaces the standard decoupled head. By optimizing the parameter-sharing mechanism between the classification and regression branches, the proposed head markedly enhances representation capability while preserving computational efficiency.
- Design of the C2DPB Backbone Module: We construct a novel C2f with Dynamic Position Bias (C2DPB) module within the backbone network. This dynamic position-bias mechanism substantially strengthens the capture of fine-grained morphological characteristics of diminutive floral targets while robustly suppressing complex background interference.
- Reconstruction of the Dynamic Neck (GDFPN): We develop a Generalized Dynamic Feature Pyramid Network (GDFPN) to reformulate the multi-scale feature fusion architecture. By enabling dynamic cross-scale feature interaction, this design effectively mitigates information loss, particularly for the segmentation of small-scale targets under drastic scale variations.
- Comprehensive Benchmarking and Edge Deployment: Extensive evaluations on a self-built dataset alongside deployment on the NVIDIA Jetson AGX Orin platform demonstrate that *Chili-Seg* outshines state-of-the-art models, delivering superior segmentation quality and an impressive real-time processing speed of 102.4 FPS.

### 1.2 Related work

Plant phenotyping aims to quantitatively characterize plant morphological, physiological, and developmental traits throughout the growth cycle, providing critical information for crop breeding, precision agriculture, and stress monitoring (Fiorani and Schurr, 2013; Yang et al., 2020). Benefiting from advances in imaging sensors and high-throughput phenotyping platforms, image-based phenotyping has become one of the most widely adopted approaches because of its non-destructive and scalable characteristics (Li et al., 2014; Paulus, 2019). Early phenotyping studies mainly relied on handcrafted visual features, including color descriptors, texture information, and geometric characteristics, for organ recognition and trait extraction. However, these methods often suffered from poor robustness under varying illumination conditions, complex backgrounds, and severe occlusions (Minervini et al., 2015). With the emergence of deep learning, convolutional neural networks (CNNs) have substantially improved feature representation capability and promoted the development of automated phenotyping systems (Mohanty et al., 2016; Kamilaris and Prenafeta-Boldu´, 2018; Jiang and Li, 2020). Mohanty et al. demonstrated that deep CNNs could achieve highly accurate plant disease recognition (Mohanty et al., 2016), while Kamilaris and Prenafeta-Boldu´ reviewed deep learning applications in agriculture and highlighted their potential in crop monitoring, yield estimation, and plant phenotyping (Kamilaris and Prenafeta-Boldu´, 2018). More recently, CNN-based methods have been widely applied to object detection, segmentation, counting, and trait extraction in high-throughput plant phenotyping (Jiang and Li, 2020).

Accurate organ segmentation is a fundamental prerequisite for quantitative phenotypic trait extraction. To obtain precise morphological information, numerous semantic and instance segmentation methods have been introduced into plant phenotyping studies. Representative architectures include Fully Convolutional Networks (FCN) (Long et al., 2015), U-Net (Ronneberger et al., 2015), DeepLab (Chen et al., 2018), and Mask R-CNN (He et al., 2017). Among them, U-Net and its variants have achieved remarkable performance in leaf segmentation, root phenotyping, and plant organ extraction owing to their encoder-decoder structure and skip-connection mechanism. Mask R-CNN further enabled instance-level segmentation of overlapping plant organs and demonstrated strong performance in complex agricultural scenes. Recently, Transformer-based architectures have introduced global contextual modeling into segmentation tasks. Vision Transformer (ViT) (Dosovitskiy et al., 2021), Swin Transformer (Liu et al., 2021), SegFormer (Xie et al., 2021), Mask2Former (Cheng et al., 2022), OneFormer (Jain et al., 2023), and the Segment Anything Model (SAM) (Kirillov et al., 2023) have achieved state-of-the-art performance across various segmentation benchmarks. Despite their impressive accuracy, these approaches typically require considerable computational resources and memory consumption, limiting their deployment on resource-constrained agricultural robotic platforms.

To meet the real-time requirements of intelligent agricultural systems, one-stage detection and segmentation frameworks have attracted increasing attention. Among them, the YOLO (You Only Look Once) family has become one of the most influential visual perception frameworks due to its favorable balance between detection accuracy and computational efficiency (Redmon et al., 2016; Redmon and Farhadi, 2018). Continuous architectural evolution has led to the development of YOLOv4 (Bochkovskiy et al., 2020), YOLOX (Ge et al., 2021), YOLOv6 (Li et al., 2022), YOLOv7 (Wang et al., 2023), YOLOv8 (Jocher et al., 2023), YOLOv9 (Wang et al., 2024b), YOLOv10 (Wang et al., 2024a), and YOLO11 (Jocher and Qiu, 2024), which progressively improved feature extraction capability, localization accuracy, and deployment efficiency. The YOLO series has been extensively applied to agricultural vision tasks such as fruit detection, flower recognition, crop monitoring, pest identification, and yield estimation (Mirhaji et al., 2021; Wang et al., 2022b; Moreira et al., 2025; Dong et al., 2025; Kuang et al., 2026k). Furthermore, segmentation-oriented variants including YOLOv5-Seg, YOLOv8-Seg, and YOLO11n-seg integrate object detection and mask prediction within a unified architecture (Jocher, 2020; Jocher et al., 2023; Jocher and Qiu, 2024), significantly reducing computational complexity compared with traditional two-stage segmentation methods while maintaining competitive segmentation performance. Nevertheless, accurately segmenting chili flowers remains challenging because of their small size, dense distribution, irregular morphology, and severe occlusion by surrounding foliage. In addition, quantitative phenotyping requires not only accurate localization but also precise boundary delineation. Therefore, developing a lightweight and high-accuracy YOLO-based segmentation framework capable of preserving fine-grained floral features is essential for reliable chili flower phenotyping and intelligent crop monitoring.

## 2 Materials and methods

### 2.1 Image acquisition and dataset construction

The image acquisition workflows for this study were conducted at the chili experimental research station of the College of Horticulture, Hunan Agricultural University (28°11′N, 113°04′E). To construct a high-quality, high-resolution dataset tailored for the fine-grained recognition and developmental stage classification of chili flowers and buds, the “8214upup” mutant line, characterized by its distinct uprightgrowth habit, was selected as the experimental material. Image data were captured utilizing a Canon R50 high-resolution digital camera during the peak flowering phenophase from June to August 2025. To mitigate the adverse effects of ambient illumination fluctuations and ensure the plants were in an optimal physiological state for observation, data collection was strictly constrained to diurnal intervals with stable ambient light, specifically 06:00–11:00 and 15:00–17:00.

In terms of sampling strategy, a multi-angle continuous observation protocol was employed to enrich the descriptive capability of the dataset across dimensions such as target morphology, scale variations, and complex field backgrounds. By systematically adjusting the camera’s pitch angles, horizontal orientations, and focal lengths, diverse floral structures across multiple developmental stages were comprehensively captured, thereby enhancing the phenotypic diversity of the repository. The field phenotypic image acquisition workflow for chili flowers is illustrated in **Figure 1**.

**Figure 1:**
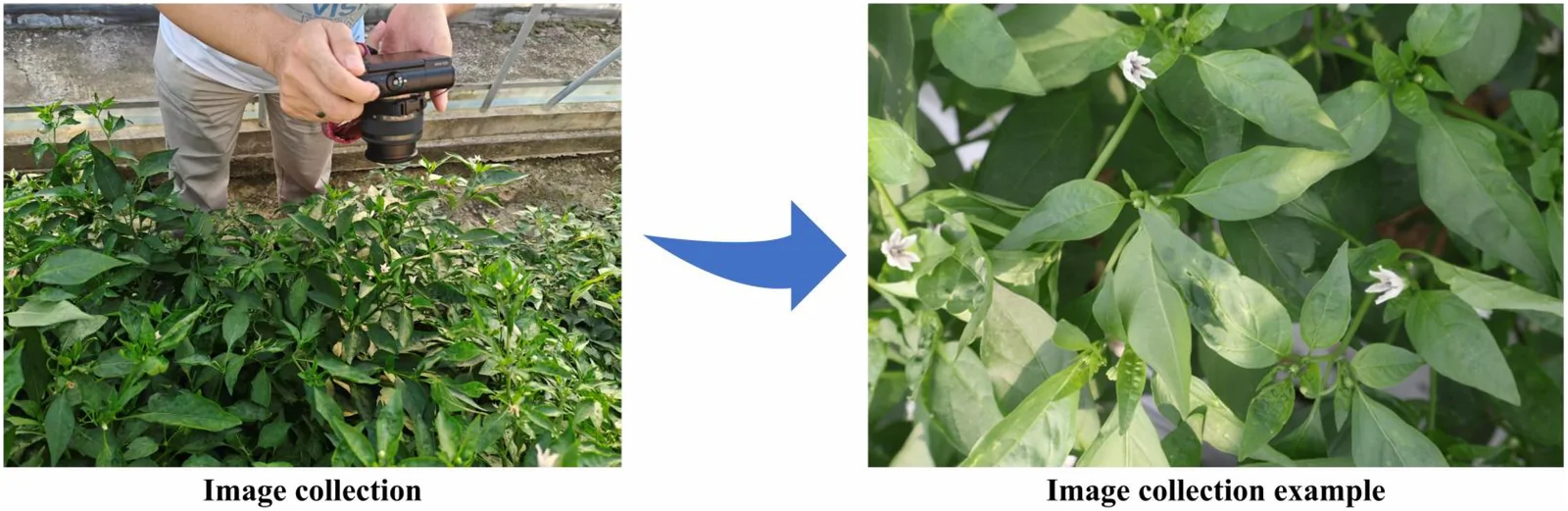
Segmentation image acquisition of chili flowers.

After manual screening to remove low-quality samples with severe motion blur or heavy occlusion, a total of 1,875 high-resolution original chili flower images were retained. All raw images were randomly and evenly partitioned via Python scripts, yielding 636 original training images, 619 original validation images and 620 original test images. To enrich the diversity of training samples, multi-dimensional hybrid offline data augmentation was exclusively applied to the 636 divided raw training images. Cyclic resampling combined with multiple transformation strategies was adopted, including geometric transformations (random rotation and translation), optical perturbations (brightness and contrast adjustments), as well as Gaussian noise and salt-and-pepper noise simulation. Finally, the training subset was expanded to 4,089 samples. No augmentation operations were conducted on the validation and test sets throughout the experiment, which only kept unmodified raw images with fixed quantities of 619 and 620 respectively. The total number of samples in the complete dataset is 5,328 (see **Figure 2**). This pipeline greatly expands the feature distribution space of training samples and effectively improves the model’s adaptability to multi-scale targets and volatile field illumination conditions. Finally, the X-AnyLabeling annotation toolkit was utilized to draw precise bounding boxes and label segmentation masks for all original images.

**Figure 2:**
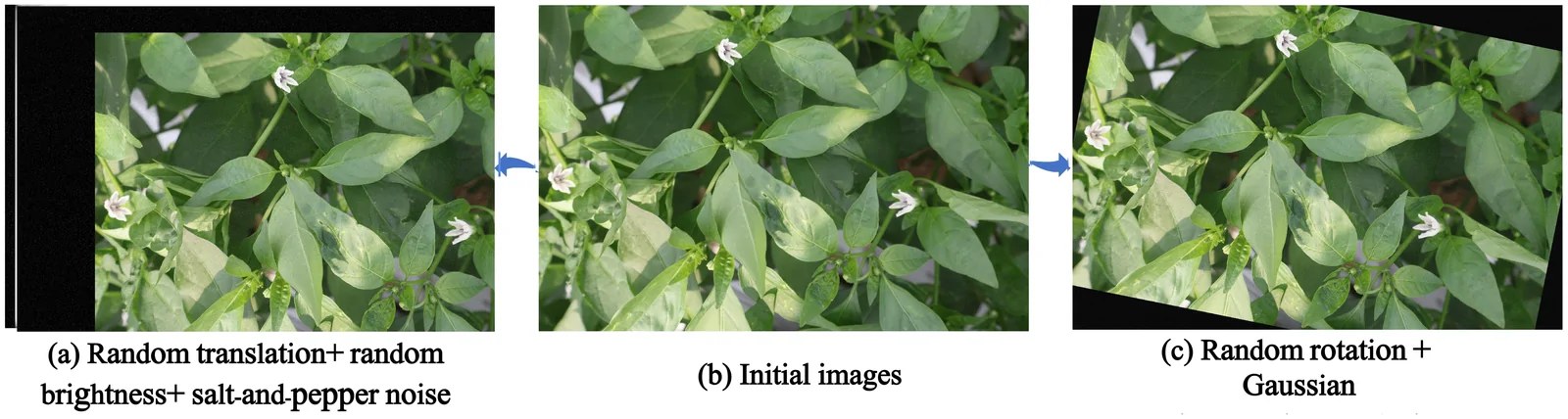
Examples of data augmentation for the chili flower dataset: **(a)** random translation, random brightness adjustment, and salt-and-pepper noise; **(b)** initial image; **(c)** random rotation, Gaussian noise, and random occlusion.

### 2.2 Overall technological route

To overcome the visual perception challenges encountered in unstructured field environments, including the diminutive scale of chili flowers, complex backgrounds, and frequent foliar occlusions, this study proposes Chili-Seg. This advanced instance segmentation model incorporates a multi-scale dynamic feature fusion network and an engineered efficient decoupling mechanism. The comprehensive research paradigm encompasses dataset construction, architectural optimization, feature visualization analysis, and edge platform deployment verification, establishing a closed-loop workflow from raw data acquisition to field application. The overall technical pipeline is schematically illustrated in **Figure 3**.

**Figure 3:**
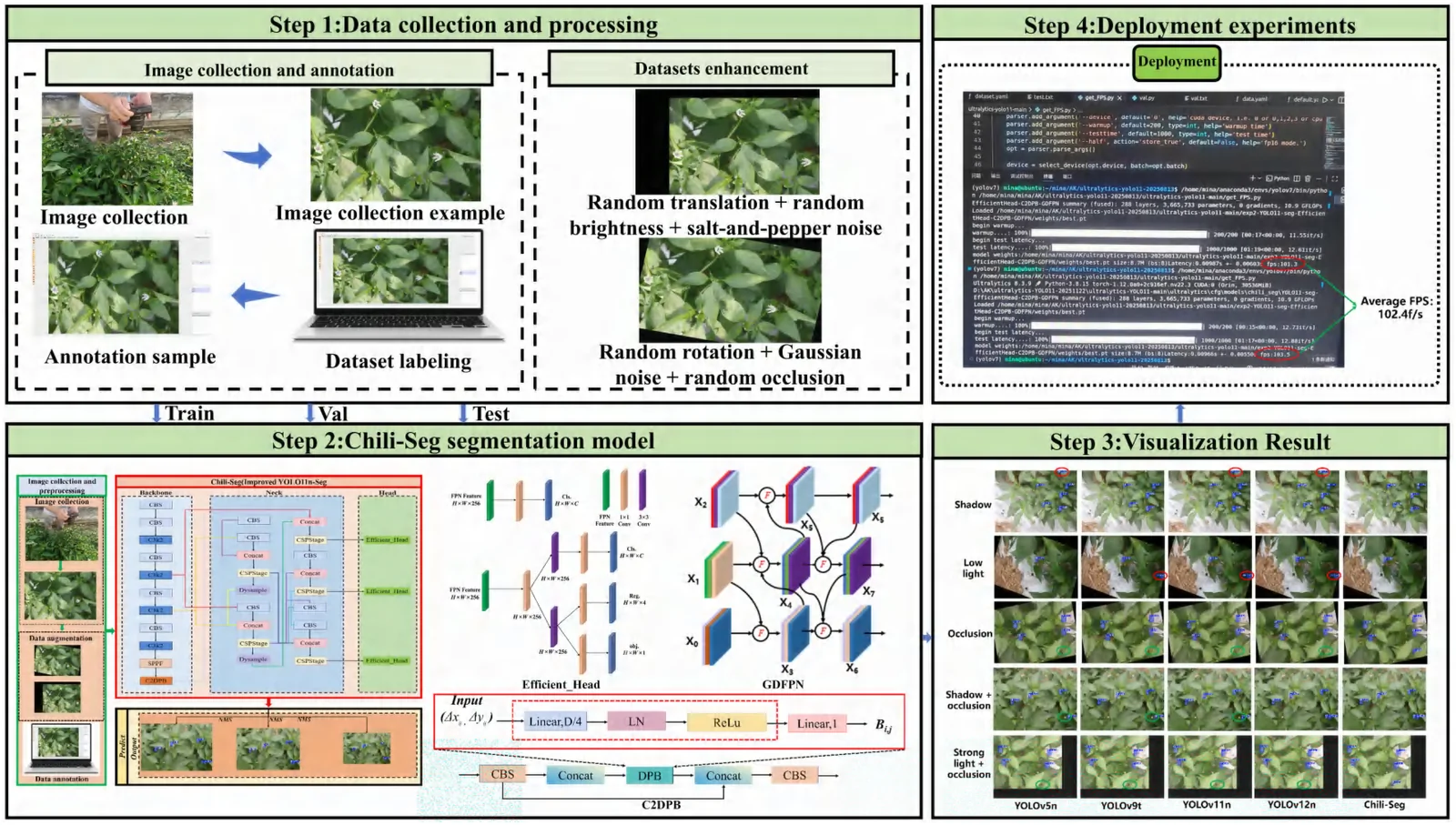
Overall technological route of the proposed chili flower segmentation framework.

The methodology is systematized into four consecutive operational phases:

- Step1: Image Acquisition and Phenotypic Dataset Standardization. Chili flower images are comprehensively captured via a multi-angle observation protocol across diverse growth stages, volatile illumination conditions, and varying occlusion degrees. Following an initial quality control screening to eliminate substandard samples, a refined phenotypic repository is established. To enrich data diversity and fortify model generalization, a hybrid data augmentation pipeline is executed. Concurrently, professional annotation toolkits are deployed to generate instance-level bounding boxes and pixel-level masks, which provides a robust data foundation for training and performance evaluation.
- Step2: Structural Engineering and Optimization of Chili-Seg. Built upon the YOLO11n-seg baseline framework, targeted architectural modifications are executed across three critical components consisting of the detection head, the backbone, and the neck network. First, to address the insufficient representation of classification and localization information in conventional detection heads under small-target scenarios, an *Efficient Head* is integrated to simultaneously elevate detection and segmentation accuracy. Second, to mitigate the attenuation of fine-grained phenotypic characteristics amidst complex field backgrounds, a novel *C2f with Dynamic Position Bias (C2DPB)* module is designed to replace the native C2PSA structure. This dynamic position-bias mechanism significantly strengthens the feature representation of critical floral zones such as corolla boundaries and stamens. Third, to counteract the information loss caused by drastic variations in target resolution, a *Generalized Dynamic Feature Pyramid Network (GDFPN)* is formulated. By leveraging DySample dynamic upsampling and adaptive weighted fusion mechanisms, this structure substantially amplifies cross-scale feature interactions and sharpens multi-scale target representations.
- Step3: Model Training and Feature Response Analysis. The optimized network undergoes joint optimization for both object detection and instance segmentation tasks under a standardized hyperparameter configuration, yielding unified target localization and boundary contour extraction. Furthermore, feature heatmaps are computed to visually analyze the spatial regions of interest and intensity of feature responses, thereby providing interpretability and empirically validating the mechanistic efficacy of each modified module under intricate outdoor scenes.
- Step4: Edge Computing Deployment and Field Validation. The optimized *Chili-Seg* framework is physically compiled and deployed onto the NVIDIA Jetson AGX Orin edge computing platform. Rigorous benchmarking is conducted to quantify its real-time inference latency and computational resource footprints, which allows for a comprehensive evaluation of its operational efficiency and viability under hardware-constrained environments. This final phase establishes the foundational technical threshold for subsequent integration into intelligent breeding robotics and automated agricultural machinery.

## 3 Chili flower phenotype recognition and segmentation algorithm and its improvements

### 3.1 YOLO11n-seg baseline architecture

YOLO11 is a single-stage real-time visual perception framework whose architecture comprises an input stage, a backbone, a feature-fusion neck, and an anchor-free decoupled prediction head (Jocher and Qiu, 2024). The framework integrates optimized C3k2 and C2PSA modules based on Position-Sensitive Attention (PSA) into the backbone network to strengthen the representation of fine-grained features. Concurrently, a bidirectional feature fusion topology consisting of a Feature Pyramid Network (FPN) and a Path Aggregation Network (PAN) is utilized to achieve deep integration of high-level semantic abstractions with shallow, high-resolution spatial information. On this basis, the decoupled detection head converts the blended features into precise bounding box regression coordinates and instance masks, striking a robust balance between computational inference velocity and detection accuracy. By leveraging its multi-scale feature capturing capacity and focused attention mechanisms, YOLO11n-seg delivers high-throughput, pixel-level visual perception, which establishes a dependable sensing foundation for intelligent crop phenotypic screening and the precise visual servoing of robotic pollination arms.

However, when applied to the extraction of chili flower phenotypes under complex field scenarios, the baseline YOLO11n-seg framework encounters distinct technical bottlenecks that limit its efficacy. First, after undergoing successive layers of deep convolutional downsampling, the topological edge features of diminutive chili flowers are highly susceptible to semantic information loss within the deeper layers of the network, which subsequently degrades the accuracy of phenotypic instance segmentation. Second, the native feature extraction and fusion mechanisms exhibit insufficient robustness against feature confusion induced by intense leaf reflections and severe structural occlusions, frequently leading to false segmentations and missed detections. Finally, the parameter footprint of the standard model remains redundant for computationally constrained agricultural edge terminals. Consequently, it is imperative to implement targeted structural adaptations and enhancements on the baseline YOLO11n-seg network based on the agronomic and biological characteristics of chili flowers, thereby satisfying the rigorous segmentation precision required for intelligent breeding phenotype identification and robotic pollination systems. The optimized architectural topology of the proposed model is comprehensively illustrated in **Figure 4**.

**Figure 4:**
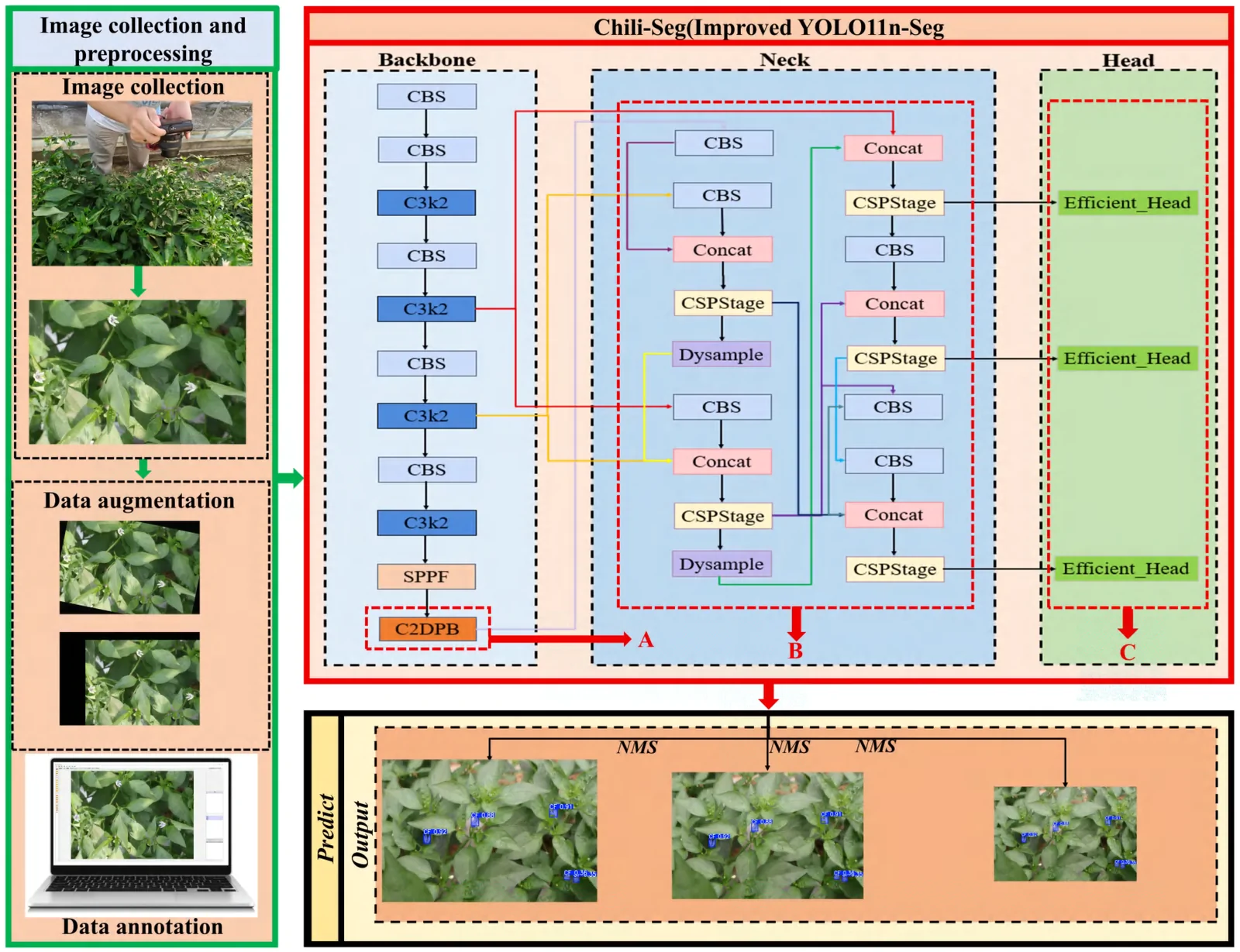
Architecture of the proposed Chili-Seg model. The red dashed boxes and arrows highlight the three major structural improvements over the original YOLO11n-seg framework: **(A)** the Efficient Head for efficient detection and instance segmentation prediction, **(B)** the C2DPB module for enhanced spatial feature representation, and **(C)** the GDFPN neck for improved multi-scale feature fusion.

In conventional YOLO architectures, the detection head typically employs a coupled structure where the tasks of object classification, bounding box regression, and mask segmentation share a monolithic feature extraction branch. However, distinct predictive tasks exhibit significantly disparate requirements for feature perception. Localization and segmentation tasks depend heavily on localized spatial details and edge textural profiles, whereas classification tasks favor global semantic abstractions and spatial invariant attributes. For chili flower phenotypic analysis, the targets are characterized by a diminutive scale, dense spatial distribution, and frequent foliar occlusions within unstructured field environments. Under these adverse conditions, conventional coupled heads are highly susceptible to feature conflicts when processing heterogeneous tasks simultaneously, making it difficult for the model to reconcile fine-grained segmentation with accurate classification, which ultimately compromises the overall precision of phenotypic parameter extraction. In light of these challenges, an Efficient Decoupled Head is integrated into the YOLO11n-seg architecture to segregate the feature channels of classification from those of localization and segmentation, thereby eliminating mutual interference in multi-task learning and enhancing the network’s capacity to parse the complex phenotypic traits of chili flowers. The topological structure of the Efficient Decoupled Head is presented in **Figure 5**.

**Figure 5:**
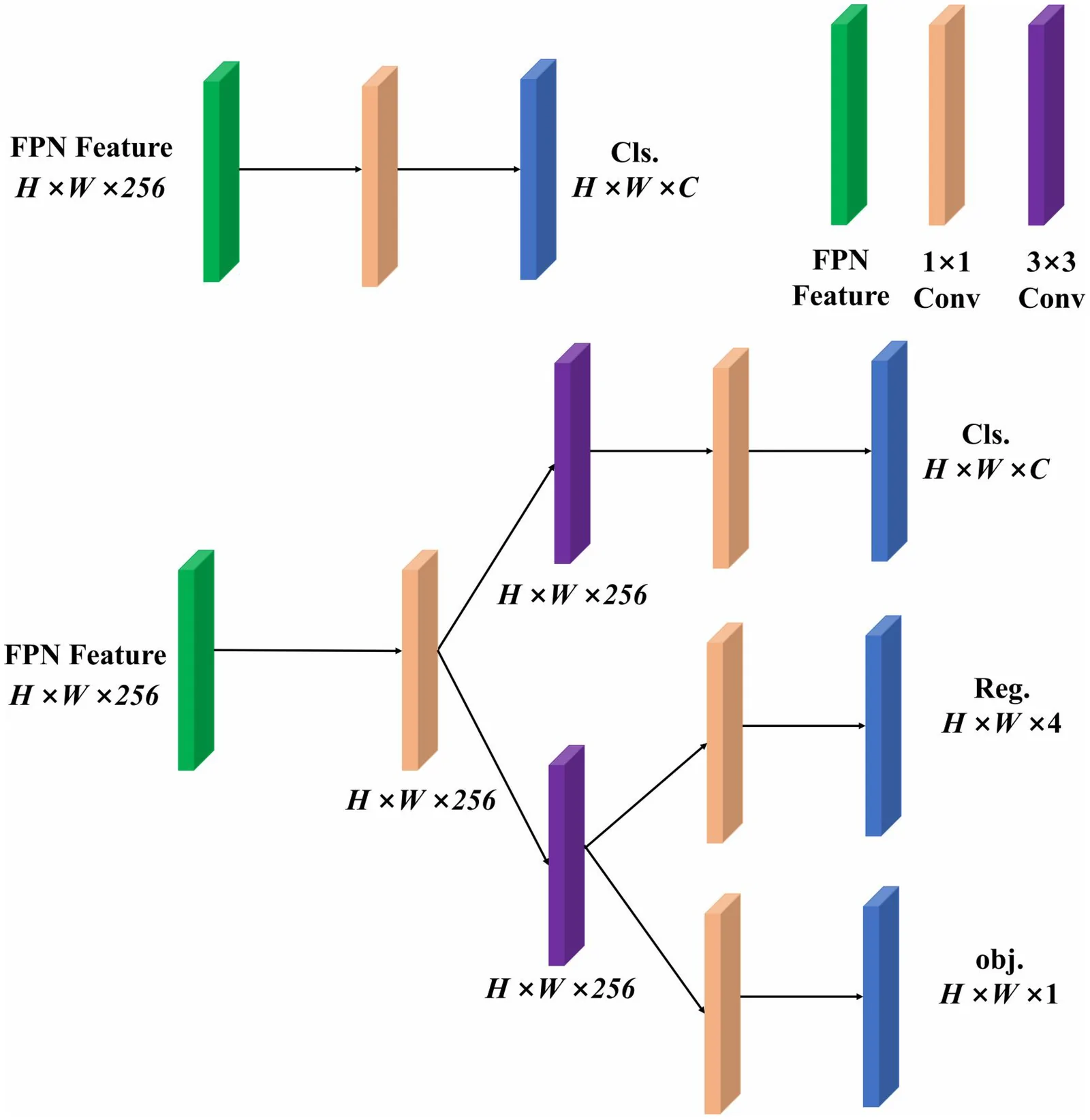
Structure of the Efficient-Head detection head. Note: The FPN serves as the input feature map, with a dimension defined as *H* × *W* ×256, where *H* and *W* represent the height and width of the feature map, respectively; and 256 denotes the number of base channels. The feature map is first processed by a 1×1 convolution layer for channel dimension adjustment, and then split into upper and lower branches. The upper branch is a single-branch classification structure, which extracts spatial features via a 3×3 convolution and finally outputs classification results with a dimension of *H* × *W* × *C*, where *C* stands for the number of object categories. The lower branch adopts a multi-task parallel structure, further divided into three sub-branches for classification, regression, and object confidence, which output feature maps of dimensions *H* × *W* × *C*, *H* × *W* ×4, and *H* × *W* ×1 respectively for category prediction, bounding box regression, and object existence judgment.

Compared with standard decoupled heads that incur prohibitive computational overhead by stacking numerous 3×3 convolutional layers in each branch, the Efficient Decoupled Head utilized in this study features a streamlined design to meet the real-time constraints of agricultural edge computing hardware while preserving high accuracy. The Efficient Decoupled Head initially receives feature maps exported from the Feature Pyramid Network (FPN) and applies a 1×1 convolutional layer for channel dimension alignment and initial feature consolidation. Subsequently, the pipeline splits into two independent pathways, where the upper branch incorporates a single 3×3 convolutional layer dedicated exclusively to target category prediction/classification (Cls). Concurrently, the lower branch extracts spatial localization features through a shared 3×3 convolutional layer before subdividing into two sub-branches for bounding box regression (Reg) and object confidence prediction (Obj). To align with the inherent configuration of YOLO11n-seg, the mask generation task is seamlessly integrated into this spatial pathway. This structural configuration, based on a hybrid channel strategy, restricts the number of intermediate 3×3 convolutional layers to one, which prevents an explosion in computational complexity caused by arbitrary channel expansion and achieves a balance between operator representation capability and hardware computational constraints. Through this architectural optimization, the model delivers richer semantic channels for classification alongside more precise spatial channels for localization and segmentation, effectively elevating the detection and segmentation accuracy of chili flower phenotypes while maintaining minimal inference latency.

### 3.2 C2DPB feature extraction unit design

Within unstructured field environments, chili flowers typically exhibit substantial morphological diversity due to the compounding effects of wind perturbations, illumination fluctuations, and changing camera viewpoints. The baseline YOLO11n-seg framework employs the C2PSA module based on PositionSensitive Attention (PSA) after the SPPF stage; the module contains stacked PSABlock units that combine multi-head self-attention and feed-forward operations (Jocher and Qiu, 2024). In the native implementation, C2PSA does not use the fixed relative-position lookup table assumed in our earlier description. The overall structure of the proposed C2DPB module is shown in **Figure 6**.

**Figure 6:**
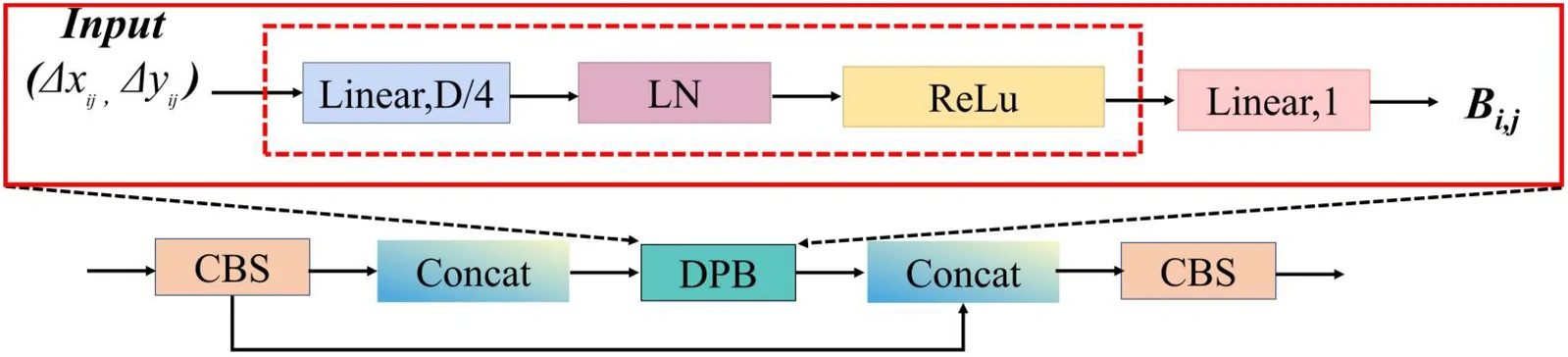
Structure of the C2DPB module.

To provide an explicit position-dependent term that can adapt to varying spatial configurations, the proposed C2DPB incorporates a Dynamic Position Bias (DPB) into the attention logits. Following the continuous relative-position formulation introduced in CrossFormer (Wang et al., 2022a), the attention operation is formulated as shown in Equation 1.

(1) $$\text{Attention}=\text{Softmax}\left(\frac{QK^{T}}{\sqrt{d}}+B\right)V$$

where *Q*, *K*, and *V* denote the Query, Key, and Value matrices, respectively, *d* is the attention scaling factor, and *B* is the position-bias term predicted from relative spatial coordinates.

To strengthen position-aware feature modeling without relying on a fixed-size discrete lookup table, this study designs the *C2f with Dynamic Position Bias* (C2DPB) feature extraction unit. The DPB generator maps the two-dimensional relative coordinate offset between tokens to a scalar bias through a Multi-Layer Perceptron (MLP), following the principle of continuous dynamic position bias used in CrossFormer (Wang et al., 2022a). This formulation allows the positional term to be computed for different feature-map sizes rather than being restricted to a predefined relative-position table. The dynamic bias is expressed as shown in Equation 2.

(2) $$B_{i,j}=\text{DPB}\left(\Delta x_{ij},\Delta y_{ij}\right)$$

Specifically, the DPB module receives the two-dimensional relative coordinate vector $\left(\Delta x_{ij},\Delta y_{ij}\right)$ as input and processes it through a sequence of non-linear transformations comprising linear mapping layers, Layer Normalization (LN), and Rectified Linear Unit (ReLU) activation functions. To guarantee computational efficiency, the dimension of the intermediate hidden layer is compressed to *D/*4, where *D* represents the embedding dimension, ultimately outputting a scalar *B_i_*,*_j_* that precisely quantifies the spatial correlation between the *i*-th and *j*-th tokens. Because the DPB operates as a continuous mapping function that is optimized end-to-end concurrently with the entire Transformer architecture, it seamlessly handles input images or feature patches of arbitrary scales. This approach entirely eliminates the clipping vulnerabilities associated with relative coordinates $\left(\Delta x_{ij},\Delta y_{ij}\right)$, enabling smooth transitions and highprecision representations of spatial position information, which establishes a rigorous foundation for the high-fidelity extraction of chili flower phenotypic parameters under dense occlusion.

### 3.3 Generalized dynamic feature pyramid network design

Within unstructured field environments, chili flowers exhibit drastic multi-scale variations and frequently present irregular morphology induced by dense foliar occlusions. The baseline YOLO11n-seg framework utilizes a Path Aggregation Feature Pyramid Network (PAFPN) as its neck architecture. Although the conventional PAFPN integrates shallow high-resolution spatial information with deep semantic abstractions through bidirectional top-down and bottom-up pathways, it encounters two prominent limitations when parsing diminutive chili flowers with intricate boundaries. First, the upsampling operations in the standard PAFPN rely heavily on nearest-neighbor interpolation, a static upsampling approach that tends to blur edge details of minute petals or buds and can introduce geometric artifacts. Second, due to the relatively rigid fusion topology, the efficiency of information exchange across disparate feature hierarchies remains restricted, making it inadequate for robust cross-scale feature alignment under severe occlusion. To surmount these theoretical bottlenecks and further elevate the model’s capacity to capture fine-grained phenotypic characteristics, this study incorporates the point-sampling-based dynamic upsampling operator DySample (Liu et al., 2023) into the Reparameterized Generalized Feature Pyramid Network (RepGFPN) design reported in DAMO-YOLO (Xu et al., 2022), thereby formulating a Generalized Dynamic Feature Pyramid Network (GDFPN) to reconstruct the neck architecture of YOLO11n-seg. The topological flow of the GDFPN feature fusion network is illustrated in **Figure 7**.

**Figure 7:**
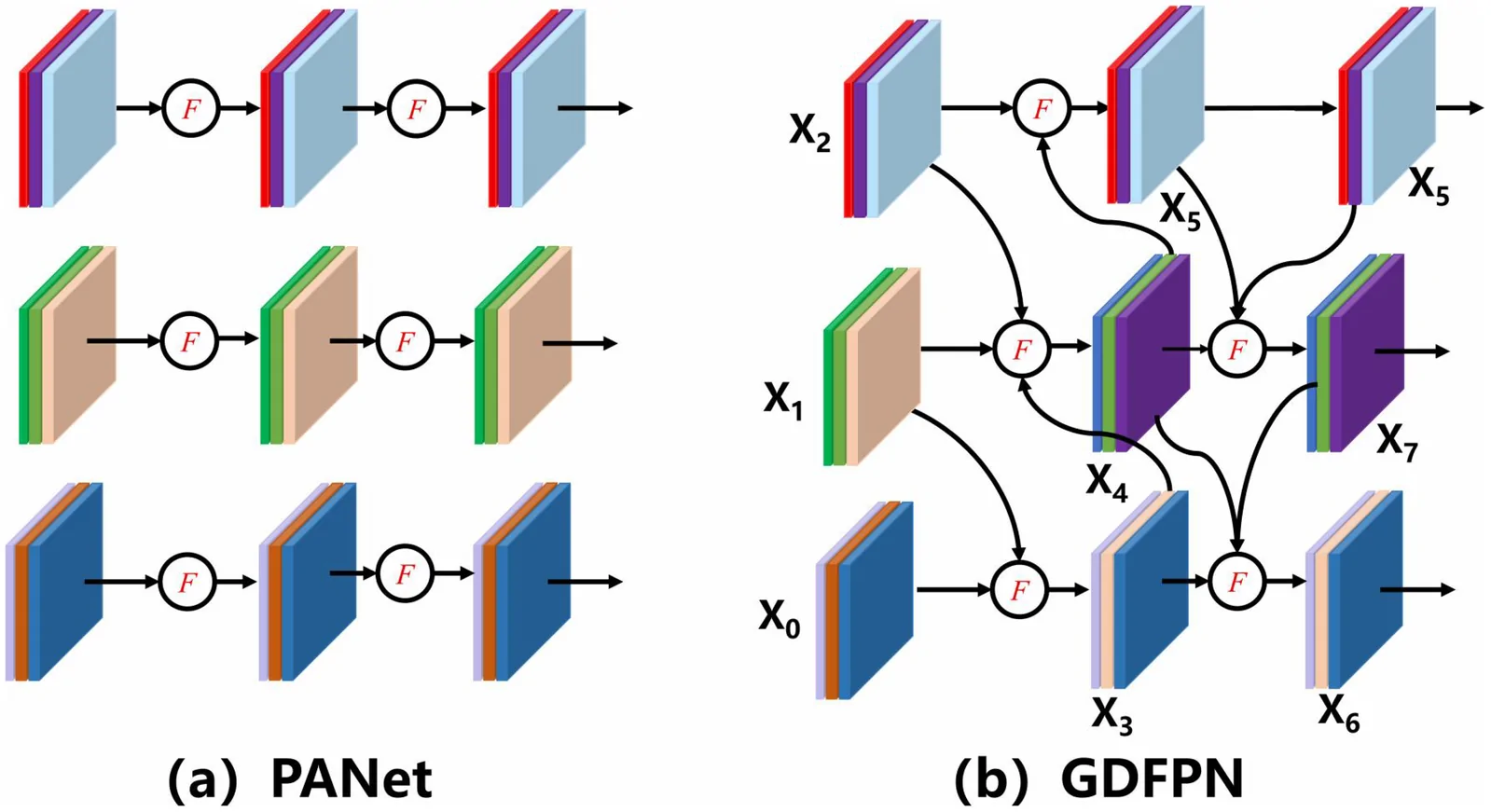
Structures of the feature fusion networks: **(a)** PANet; **(b)** the proposed GDFPN.

The structural core of the GDFPN lies in the deep coupling of a multi-stage cross-layer fusion paradigm with dynamic upsampling operations, which generates a highly perceptive multi-scale feature pyramid to supply robust representation support for chili flower detection in complex field scenarios. Letting the multi-scale input feature set exported from the backbone network be defined as $\left\{x_{0},x_{1},x_{2}\right\}$, each feature map corresponds to a specific hierarchy where the receptive field expands sequentially from shallow to deep layers. During the bottom-up feature aggregation sequence, the network discards traditional static interpolation techniques in favor of the DySample dynamic upsampling strategy. This dynamic operator adaptively optimizes the spatial distribution of sampling points based on the local texture profiles and target scales of the chili flowers and buds, achieving high-fidelity resolution expansion of the antecedent feature map *x*_3_ while preserving the structural details of minuscule targets to the maximum extent. Subsequently, the high-resolution upsampled features are densely concatenated with the deep features *x*_1_ and *x*_2_ after convolutional regularization, completing a deep integration of cross-layer information.

This dynamic fusion mechanism is recursively executed during the inference computations of the higher dimensional feature *x*_5_, thereby constructing a multi-stage, cross-scale, closed-loop feature propagation pathway. By leveraging this advanced multi-stage paradigm, the GDFPN facilitates comprehensive interaction and mutual complementation between shallow spatial-geometric profiles and deep global contextual information across diverse feature granularities. Consequently, the network encourages its receptive field to focus on the target regions, which significantly mitigates the feature attenuation typically associated with conventional upsampling processes, sustains the high-fidelity extraction of fine-grained crop phenotypic attributes, and harvests rich contextual semantics to substantially enhance overall instance segmentation performance.

### 3.4 Experimental platform and evaluation metrics

#### 3.4.1 Experimental platform

To ensure the objectivity and reproducibility of the performance evaluation for the chili flower phenotypic analysis model, all architectural re-engineerings, ablation studies, and baseline comparative benchmarks in this study were deployed on a unified computing workstation. The hardware infrastructure comprised an Intel Core i5-13600KF processor and an NVIDIA GeForce RTX 3060 Ti graphics processing unit (GPU) with 8 GB of Video RAM (VRAM), utilizing the Compute Unified Device Architecture (CUDA) 11.8 parallel computing architecture to accelerate mask generation and gradient descent processes. The software environment was established on the Windows 11 operating system, using Python 3.8 as the core development language, with network construction and algorithmic optimization executed under the PyTorch deep learning framework.

During the model training phase, all comparative experiments strictly adhered to the principle of controlled variables to prevent the networks from overfitting to specific field illumination conditions or localized background noise. Crucially, all models were trained from scratch without utilizing pre-trained weights to rigorously verify the feature extraction modules’ intrinsic semantic perception capabilities for chili flower phenotypes. The network optimization was performed using the Stochastic Gradient Descent (SGD) optimizer, coupled with a Cosine Annealing learning rate scheduling strategy to facilitate convergence toward the global optimum. All other hyperparameters were maintained at their default configurations, and the uniform hyperparameter settings enforced across all experiments are detailed in **Table 1**.

**Table 1:** Hyperparameter configurations utilized during the model training phase.

Training parameter	Value or type
Input image size	640×640×3
Optimizer	SGD
Optimizer momentum	0.937
Optimizer weight decay rate	0.0005
Initial learning rate	0.01
Final learning rate	0.01
Batch size	8
Number of classes (*nc*)	1
Total epochs	200

#### 3.4.2 Evaluation metrics

Given that chili flower phenotypic analysis involves both object localization and pixel-level instance delineation, this study evaluates model performance from two complementary perspectives: bounding-box detection and instance segmentation. Precision (*P*), Recall (*R*), Average Precision (*AP*), and mean Average Precision (*mAP*) are calculated separately for bounding boxes (Detect/Box) and instance masks (Mask). In addition, Intersection over Union (IoU), mean Intersection over Union (*mIoU*), and mean Pixel Accuracy (*mPA*) are used for the semantic segmentation models involved in the comparative experiments. The corresponding metrics are defined in Equations 3–8.

(3) $$P=\frac{C_{TP}}{C_{TP}+C_{FP}}\times 100\%$$

(4) $$R=\frac{C_{TP}}{C_{TP}+C_{FN}}\times 100\%$$

(5) $$AP=\int_{0}^{1}P\left(R\right)\;dR$$

(6) $$mAP=\frac{{\sum_{i=1}^{n}AP}_{i}}{n}$$

(7) $$mIoU=\frac{1}{k}\sum_{i=1}^{k}IoU_{i}\times 100\%$$

(8) $$mPA=\frac{1}{k}\sum_{i=1}^{k}PA_{i}$$

Precision (*P*) represents the proportion of correctly predicted chili flower instances among all positive predictions, whereas Recall (*R*) measures the proportion of ground-truth chili flowers that are successfully identified. Here, *TP*, *FP*, and *FN* denote true-positive, false-positive, and false-negative predictions, respectively. These metrics are calculated separately for the detection and instance segmentation branches.

For the detection results, the Box metrics are calculated according to the overlap between predicted bounding boxes and ground-truth bounding boxes, and therefore mainly reflect whether a chili flower is correctly detected and accurately localized. In contrast, the Mask metrics are calculated according to the overlap between predicted instance masks and ground-truth masks, and therefore evaluate the pixel-level quality of flower-region delineation and contour fitting. Consequently, a flower may be correctly detected at the bounding-box level while still exhibiting an inaccurate instance mask; therefore, high Box performance does not necessarily imply equally accurate Mask boundaries.

For both Box and Mask evaluation, *mAP*_50_ denotes the mean Average Precision at an IoU threshold of 0.50, while *mAP*_50–95_ represents the average mAP over IoU thresholds from 0.50 to 0.95 with a step size of 0.05. Compared with *mAP*_50_, *mAP*_50–95_ provides a more stringent evaluation of localization and segmentation quality because it requires predictions to remain accurate under progressively higher overlap criteria. For Box evaluation, a higher *mAP*_50–95_ indicates more accurate bounding-box localization; for Mask evaluation, it reflects better agreement between the predicted instance masks and the ground-truth flower contours.

The Average Precision (*AP*) is defined as the area under the Precision–Recall curve, and *mAP* is obtained by averaging the AP values across all target categories. Since only one chili flower category is considered in this study, *n* = 1.

The *mIoU* and *mPA* metrics are used specifically for semantic segmentation evaluation. *mIoU* measures the average overlap between predicted semantic regions and ground-truth regions across classes, whereas *mPA* measures the average pixel-wise classification accuracy. These semantic segmentation metrics belong to a different evaluation framework from instance-level Mask *mAP* and therefore should not be interpreted as directly equivalent or numerically interchangeable with the Mask metrics reported for Chili-Seg.

Model complexity and inference efficiency are further characterized by the number of parameters (Params), floating-point operations (FLOPs), giga floating-point operations (GFLOPs) where applicable, and frames per second (FPS).

## 4 Results and discussion

### 4.1 Ablation studies

Ablation experiments were systematically designed to dissect the network structure, allowing for a scientific analysis of the independent contributions and synergistic effects of each modified module on the overall performance of the model. To address the challenges of small target dimensions, dense foliar occlusions, and volatile illumination perturbations characteristic of chili flowers in natural fields, this study utilized the YOLO11n-seg model as the baseline framework. The ablation matrix revolved around three core structural modifications: Module A (the incorporation of the Efficient Decoupled Head), Module B (the integration of the C2DPB feature extraction unit), and Module C (the construction of the Generalized Dynamic Feature Pyramid Network, GDFPN).

The ablation results presented in **Table 2** demonstrate the individual and joint contributions of the proposed modules to chili flower detection and instance segmentation. The baseline YOLO11n-seg achieved a detection Precision of 95.1%, a Recall of 80.6%, an *mAP*_50_ of 89.4%, and an *mAP*_50–95_ of 76.1%. For the segmentation task, the corresponding Precision, Recall, *mAP*_50_, and *mAP*_50–95_ were 94.5%, 80.4%, 88.7%, and 63.4%, respectively.

**Table 2:** Quantitative ablation results of the modified components based on the Chili-Seg architecture.

Models	A	B	C	Detect (%)				Mask (%)			
				*P*	*R*	*mAP*_50_	*mAP*_50–95_	*P*	*R*	*mAP*_50_	*mAP*_50–95_
YOLO11n-seg(Baseline)	×	×	×	95.1	80.6	89.4	76.1	94.5	80.4	88.7	63.4
YOLO11n-seg+A	✓	×	×	95.5	81	89.7	76.1	94.9	**80.6**	88.8	63.5
YOLO11n-seg+B	×	✓	×	94.1	80.2	88.8	75.4	94	79.9	87.9	62.7
YOLO11n-seg+C	×	×	✓	95.9	80.3	90	76.9	95.5	80	89.4	64.1
YOLO11n-seg+A+B	✓	✓	×	93.9	**81.8**	89.9	76.5	94.1	**80.6**	88.9	63.4
Chili-Seg (YOLO11n-seg+A+B+C)	✓	✓	✓	**96.8**	80.6	**90.6**	**77.9**	**96.6**	80.4	**89.8**	**64.7**
Models			Params(M)			FLOPs(G)			FPS(f/s)		
YOLO11n-seg(Baseline)			2.83			9.6			194.3		
YOLO11n-seg+A			2.56			8.4			205.8		
YOLO11n-seg+B			2.85			9.6			75.6		
YOLO11n-seg+C			3.92			11.5			63.8		
YOLO11n-seg+A+B			2.58			8.4			178.1		
Chili-Seg (Ours)			3.67			10.3			148.1		

A denotes the Efficient Head, B denotes the C2DPB module, and C denotes the GDFPN. ✓ indicates the incorporation of the corresponding module, while × denotes its exclusion.Bold values indicate the best result for each metric among the compared models/configurations.

After incorporating Module A, namely the Efficient Decoupled Head, the detection Precision increased from 95.1% to 95.5%, Recall from 80.6% to 81.0%, and *mAP*_50_ from 89.4% to 89.7%, while the segmentation *mAP*_50_ increased slightly from 88.7% to 88.8%. Meanwhile, the parameter count decreased from 2.83 M to 2.56 M, FLOPs decreased from 9.6 G to 8.4 G, and the inference speed increased from 194.3 FPS to 205.8 FPS. These results suggest that the primary contribution of the Efficient Decoupled Head is to improve computational efficiency while maintaining the original detection and segmentation performance. These results indicate that the primary contribution of the Efficient Decoupled Head is to improve computational efficiency while maintaining essentially unchanged detection and instance segmentation performance, rather than providing a substantial standalone accuracy gain. Compared with this efficient configuration, the complete Chili-Seg model increases the parameter count from 2.56 M to 3.67 M and reduces the inference speed from 205.8 FPS to 148.1 FPS, but further improves the detection and mask *mAP*_50–95_ by 1.8 and 1.2 percentage points, respectively. The larger gains under stricter IoU thresholds indicate improved localization and mask-overlap quality, demonstrating a reasonable trade-off between prediction accuracy and computational efficiency. Moreover, previous studies have commonly regarded 30 FPS as a practical threshold for real-time visual processing (Liu and Kang, 2024; Hou et al., 2020). Since Chili-Seg achieves 148.1 FPS, which is substantially higher than this commonly used realtime benchmark, the reduction in inference speed does not compromise its real-time processing capability.

When Module B was independently introduced into the baseline network, the detection Recall remained competitive at 80.2%, while the detection *mAP*_50_ and *mAP*_50–95_ reached 88.8% and 75.4%, respectively. The corresponding segmentation *mAP*_50_ and *mAP*_50–95_ were 87.9% and 62.7%. Although the overall accuracy was slightly lower than that of the baseline model, the results suggest that the C2DPB module primarily strengthens the model’s sensitivity to spatial variations and target appearance changes. The substantial decrease in inference speed to 75.6 FPS further indicates that the effectiveness of this module is more evident when combined with complementary network components.

The independent introduction of Module C produced more pronounced performance gains. Detection Precision increased to 95.9%, while the detection *mAP*_50_ and *mAP*_50–95_ improved to 90.0% and 76.9%, respectively. For the segmentation task, *mAP*_50_ increased from 88.7% to 89.4%, and *mAP*_50–95_ increased from 63.4% to 64.1%. These improvements are consistent with enhanced multi-scale feature aggregation and better preservation of discriminative structural information by GDFPN. However, this performance gain was accompanied by increased computational cost, with the parameter count rising to 3.92 M, the computational complexity increasing to 11.5 GFLOPs, and the inference speed decreasing to 63.8 FPS.

When Modules A and B were jointly employed, the detection Recall reached 81.8%, representing the highest value among all ablation configurations. In addition, the detection *mAP*_50_ increased to 89.9%, and the segmentation *mAP*_50_ reached 88.9%. Compared with the baseline model, this configuration maintained lower computational complexity at 8.4 GFLOPs while achieving an inference speed of 178.1 FPS. These results demonstrate that the Efficient Decoupled Head and C2DPB module exhibit strong complementarity, enabling improved target perception and reducing missed detections in complex field environments.

By integrating all proposed modules, the final Chili-Seg model achieved the best overall performance. Detection Precision increased to 96.8%, while the detection *mAP*_50_ and *mAP*_50–95_ reached 90.6% and 77.9%, respectively. For the segmentation task, Precision increased to 96.6%, while *mAP*_50_ and *mAP*_50–95_ reached 89.8% and 64.7%, respectively. Compared with the baseline model, the final configuration improved detection *mAP*_50_ by 1.2 percentage points and detection *mAP*_50–95_ by 1.8 percentage points. The segmentation *mAP*_50_ and *mAP*_50–95_ increased by 1.1 and 1.3 percentage points, respectively. Although the parameter count and computational complexity increased to 3.67 M and 10.3 GFLOPs, respectively, the model maintained an inference speed of 148.1 FPS, which remains fully adequate for real-time deployment on agricultural robotic platforms.

Considering that several performance gains obtained by Chili-Seg are relatively modest, an additional repeated-training experiment was conducted to examine whether the reported performance is sensitive to random initialization and stochastic training variation. The final Chili-Seg configuration was independently trained using five random seeds (0, 10, 42, 123, and 3407). For all repeated experiments, the dataset split, model architecture, training hyperparameters, number of epochs, and evaluation protocol were kept unchanged. The detailed results are summarized in **Table 3**, while the corresponding mean and standard deviation are visualized in **Figure 8**.

**Table 3:** Repeated-training results of Chili-Seg under five different random seeds.

Seed	Detection (%)				Mask (%)			
	*P*	*R*	*mAP*_50_	*mAP*_50–95_	*P*	*R*	*mAP*_50_	*mAP*_50–95_
Default (0)	96.8	80.6	90.6	77.9	96.6	80.4	89.8	64.7
10	95.4	81.2	90.2	76.7	95.1	80.4	89.4	64.4
42	96.7	79.7	90.2	77.2	96.4	79.5	89.5	64.2
123	96.3	79.5	90.0	76.5	95.9	79.3	89.1	63.9
3407	96.1	80.2	90.7	77.0	95.9	80.1	90.1	64.3
Mean	96.26	80.24	90.34	77.06	95.98	79.94	89.58	64.30
Std	0.56	0.69	0.30	0.54	0.58	0.51	0.38	0.29

**Figure 8:**
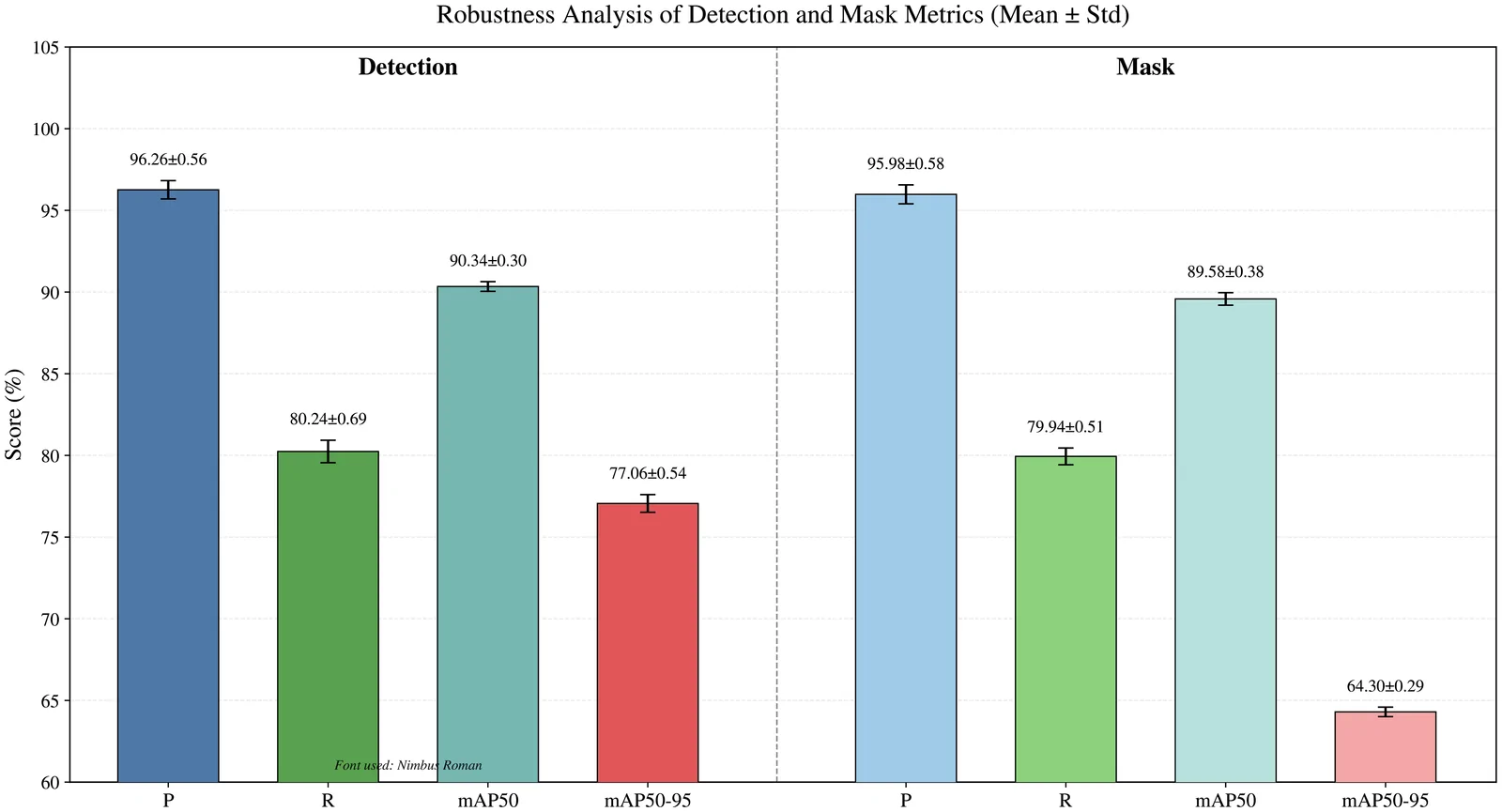
Repeated-training stability analysis of Chili-Seg under five different random seeds. The bars represent the mean values of the Detection and Mask metrics obtained from five independent training runs, while the error bars denote the corresponding standard deviations.

As shown in **Table 3**, Chili-Seg maintains relatively stable detection and instance segmentation performance across the five independent training runs. For detection, the mean Precision, Recall, *mAP*_50_, and *mAP*_50–95_ are 96.26%, 80.24%, 90.34%, and 77.06%, respectively, with corresponding standard deviations of 0.56, 0.69, 0.30, and 0.54 percentage points. For Mask prediction, the corresponding mean values are 95.98%, 79.94%, 89.58%, and 64.30%, with standard deviations of 0.58, 0.51, 0.38, and 0.29 percentage points.

The error-bar visualization in **Figure 8** further shows that the fluctuations of the major performance metrics are limited across random seeds. All reported standard deviations remain below 0.7 percentage points, with particularly small variations for detection *mAP*_50_ (0.30), Mask *mAP*_50_ (0.38), and Mask *mAP*_50–95_ (0.29). These results indicate that the final Chili-Seg configuration exhibits stable and reproducible performance rather than relying on a particular favorable random initialization.

Furthermore, the repeated-run mean detection *mAP*_50_ and *mAP*_50–95_ values of 90.34% and 77.06% remain higher than the corresponding single-run YOLO11n-seg baseline values of 89.4% and 76.1%. A similar trend is observed for instance segmentation: the repeated-run mean Mask *mAP*_50_ and *mAP*_50–95_ values are 89.58% and 64.30%, compared with 88.7% and 63.4% for the baseline. Thus, although the magnitude of improvement varies slightly among random seeds, the overall performance trend of Chili-Seg remains consistent.

Because repeated multi-seed experiments were not conducted for every intermediate ablation configuration, this stability analysis is used to demonstrate the reproducibility of the final framework and should not be interpreted as formal statistical proof that every individual increment is exclusively attributable to one architectural modification. Accordingly, the conclusions are expressed in terms of consistent and reproducible performance trends rather than statistical significance.

Overall, the ablation study demonstrates that the three proposed modules contribute from different perspectives. The Efficient Decoupled Head primarily improves computational efficiency while maintaining essentially unchanged detection and segmentation performance, the C2DPB module provides complementary target-sensitive spatial representation, and the GDFPN contributes to improved multi-scale feature interaction and stricter-IoU prediction quality. Together with the repeated-training stability results, these experiments indicate that the final Chili-Seg configuration provides a reproducible improvement in overall detection and instance segmentation performance while maintaining favorable computational efficiency.

### 4.2 Comparative experiments

#### 4.2.1 Comparative benchmarking of diverse detection heads

To systematically investigate the empirical impacts of varying detection head topologies on the performance of the chili flower phenotypic network, and to validate the architectural superiority of the selected component, a parallel comparative study was conducted. The original detection head of the baseline YOLO11n-seg framework was replaced with several state-of-the-art head structures, specifically Localization Quality Estimation (LQE), Lightweight Asymmetric Detection Head (LADH), Lightweight Shared Convolutional Detection Head (LSCD), Lightweight Shared Convolution with Separated Batch Normalization Detection Head (LSCSBD), Lightweight Shared Detail-Enhanced Convolutional Detection Head (LSDECD), and the deployed Efficient head.

The quantitative comparison results in **Table 4** demonstrate that different detection heads exhibit distinct performance characteristics in chili flower detection and segmentation tasks. Among the compared methods, LQE and LSCD achieved the highest detection Precision of 97.0%, indicating strong confidence in positive predictions. However, their Recall values decreased to 79.3%, suggesting that improvements in Precision were accompanied by a reduction in target coverage. Similar trends were observed for LADH and LSDECD, whose Recall values remained below those of the baseline model.LQE and LSCD achieved the highest detection Precision of 97.0%, indicating strong confidence in positive predictions. However, their Recall values decreased to 79.3%, suggesting that improvements in Precision were accompanied by a reduction in target coverage. Similar trends were observed for LADH and LSDECD, whose Recall values remained below those of the baseline model.

**Table 4:** Quantitative performance comparison of the chili flower phenotypic model configured with diverse detection heads.

Models	Detect (%)				Mask (%)			
	*P*	*R*	*mAP*_50_	*mAP*_50–95_	*P*	*R*	*mAP*_50_	*mAP*_50–95_
YOLO11n-seg	95.1	80.6	89.4	76.1	94.5	80.4	88.7	63.4
LQE	**97.0**	79.3	89.7	76.3	**96.7**	78.7	88.7	63.3
LADH	95.9	80.2	89.1	75.9	95.2	79.6	88.2	63.0
LSCD	**97.0**	79.3	**89.8**	**76.4**	96.5	78.9	88.7	63.7
LSCSBD	94.5	**81.4**	89.5	76.1	95.8	79.8	88.4	**64.3**
LSDECD	95.3	79.7	89.4	76.0	95.0	79.5	88.4	63.0
Efficient (Ours)	95.5	81.0	89.7	76.1	94.9	**80.6**	**88.8**	63.5

Bold values indicate the best result for each metric among the compared models/configurations.

In contrast, LSCSBD achieved the highest detection Recall of 81.4%, exceeding the baseline by 0.8 percentage points. Nevertheless, its Precision decreased to 94.5%, which resulted in only marginal improvements in overall detection accuracy. These results indicate that different head architectures emphasize different aspects of the detection task, leading to varying trade-offs between Precision and Recall.

The proposed Efficient Head achieved a more balanced performance across both detection and segmentation tasks. Compared with the baseline YOLO11n-seg, detection Precision increased from 95.1% to 95.5%, while Recall increased from 80.6% to 81.0%. The detection *mAP*_50_ reached 89.7%, which was comparable to the best-performing alternatives. In the segmentation task, the proposed head achieved the highest Recall of 80.6% and the highest mask *mAP*_50_ of 88.8%. Moreover, the mask *mAP*_50–95_ increased from 63.4% to 63.5%, indicating a consistent improvement in segmentation quality under different IoU thresholds.

Although LSCD achieved slightly higher detection *mAP*_50_ and *mAP*_50–95_ values, and LSCSBD obtained the highest Recall and mask *mAP*_50–95_, the proposed Efficient Head maintained competitive performance across all evaluation metrics without introducing obvious performance degradation in either detection or segmentation tasks. This result suggests that the Efficient Head provides a favorable balance between classification and localization learning, contributing to more stable feature representation for chili flower phenotyping under complex field conditions.

#### 4.2.2 Comparative benchmarking of diverse backbone attention modules

To systematically investigate the empirical impacts of substituting alternative attention-based components into the backbone network on chili flower phenotypic detection and instance segmentation, a parallel benchmark comprising seven comparative configurations was engineered based on the integration of the Efficient Decoupled Head. The original spatial attention component was systematically replaced with various mainstream topologies, including C2ASSA (an alternative attention-module variant), C2BRA incorporating Bi-Level Routing Attention (BRA), C2CGA incorporating Cascaded Group Attention (CGA), C2DA incorporating Deformable Attention (DA), CMSLA (an alternative attention-module variant), and the designed C2DPB module.

The quantitative comparison results in **Table 5** illustrate the influence of different attention mechanisms on chili flower detection and segmentation performance. Compared with the baseline configuration equipped with the Efficient Head, most attention modules improved specific evaluation metrics but failed to achieve consistent gains across all tasks.

**Table 5:** Quantitative performance comparison of the chili flower phenotypic model configured with diverse backbone attention mechanisms.

Models	Detect (%)				Mask (%)			
	*P*	*R*	*mAP*_50_	*mAP*_50–95_	*P*	*R*	*mAP*_50_	*mAP*_50–95_
YOLO11n-seg + Head (A)	95.5	81.0	89.7	76.1	94.9	**80.6**	88.8	**63.5**
C2ASSA	95.6	79.8	89.1	75.6	95.4	79.6	88.3	62.9
C2BRA	95.0	80.5	88.9	75.9	95.0	80.0	88.3	63.0
C2CGA	**96.3**	79.4	89.2	75.5	**96.3**	78.9	88.2	62.5
C2DA	94.5	80.4	89.2	75.3	94.6	79.9	88.1	62.6
CMSLA	95.8	80.2	89.3	75.9	95.1	79.7	88.2	62.6
C2DPB (Ours)	93.9	**81.8**	**89.9**	**76.5**	94.1	**80.6**	**88.9**	63.4

Bold values indicate the best result for each metric among the compared models/configurations.

Among the compared methods, C2CGA achieved the highest detection Precision of 96.3% and the highest segmentation Precision of 96.3%. However, its Recall values decreased to 79.4% and 78.9% for detection and segmentation, respectively. Similar trends were observed for C2ASSA and CMSLA, which achieved relatively high Precision but exhibited lower Recall and reduced performance under the *mAP*_50_ and *mAP*_50–95_ metrics. In contrast, C2BRA and C2DA produced performance levels comparable to those of the baseline model, but neither method yielded noticeable improvements in overall detection or segmentation accuracy.

The proposed C2DPB module achieved the highest detection Recall of 81.8%, representing an improvement of 0.8 percentage points over the baseline model. At the same time, the detection *mAP*_50_ and *mAP*_50–95_ increased from 89.7% to 89.9% and from 76.1% to 76.5%, respectively. Similar improvements were observed in the segmentation task, where the mask *mAP*_50_ increased from 88.8% to 88.9%, while the mask *mAP*_50–95_ remained competitive at 63.4%. These results indicate that C2DPB contributes to improved target coverage and overall localization performance.

Although the Precision of C2DPB was slightly lower than that of several competing attention mechanisms, it achieved the best overall balance among Recall, *mAP*_50_, and *mAP*_50–95_. Since chili flower phenotyping requires reliable detection of small and partially occluded targets, improvements in Recall and localization accuracy are particularly important. The superior performance of C2DPB under these metrics demonstrates its effectiveness in enhancing feature representation for challenging field environments.

#### 4.2.3 Comparative benchmarking of diverse feature fusion networks

To systematically evaluate the empirical impacts of varying neck feature fusion architectures on chili flower phenotypic detection and instance segmentation performance, a parallel comparative study encompassing six algorithmic configurations was executed based on the integrated Efficient Decoupled Head and the C2DPB module. The neck architecture was systematically replaced with various mainstream multi-scale feature fusion networks, including Bidirectional Feature Pyramid Network (BiFPN), BiMAFPN (a bidirectional multi-branch feature-pyramid variant), EMBSFPN (a multi-scale feature-pyramid variant), CGRFPN (a context-guided feature-pyramid variant), and the proposed GDFPN.

The quantitative comparison results in **Table 6** demonstrate the influence of different feature fusion networks on chili flower detection and segmentation performance. Although several feature fusion strategies achieved competitive Precision values, their overall performance remained limited when evaluated using comprehensive accuracy metrics.

**Table 6:** Quantitative performance comparison of the chili flower phenotypic model configured with diverse feature fusion networks.

Models	Detect (%)				Mask (%)			
	*P*	*R*	*mAP*_50_	*mAP*_50–95_	*P*	*R*	*mAP*_50_	*mAP*_50–95_
YOLO11n-seg + Head (A) + Backbone (B)	93.9	**81.8**	89.9	76.5	94.1	**80.6**	88.9	63.4
BiFPN	94.8	80.3	89.6	75.6	95.2	79.4	88.7	63.2
BiMAFPN	96.2	79.4	89.5	75.5	95.8	78.8	88.1	63.0
EMBSFPN	96.1	79.0	88.2	74.7	95.7	78.7	87.3	62.8
CGRFPN	95.1	80.0	88.8	75.1	95.0	79.2	87.6	62.8
GDFPN (Ours)	**96.8**	80.6	**90.6**	**77.9**	**96.6**	**80.4**	**89.8**	**64.7**

Bold values indicate the best result for each metric among the compared models/configurations.

Among the compared methods, BiMAFPN and EMBSFPN achieved relatively high detection Precision values of 96.2% and 96.1%, respectively. However, their Recall values decreased to 79.4% and 79.0%, and their detection *mAP*_50_ values remained below 90%. Similar trends were observed for BiFPN and CGRFPN, whose detection and segmentation metrics were consistently lower than those of the baseline configuration. In particular, the mask *mAP*_50–95_ values of all compared feature fusion networks remained below 63.2%, indicating limited improvements in fine-grained segmentation quality.

The proposed GDFPN achieved the best overall performance among all evaluated feature fusion networks. Detection Precision increased to 96.8%, while the detection *mAP*_50_ and *mAP*_50–95_ reached 90.6% and 77.9%, respectively. Compared with the baseline configuration, these values correspond to improvements of 2.9 percentage points in Precision, 0.7 percentage points in *mAP*_50_, and 1.4 percentage points in *mAP*_50–95_. Similar improvements were observed in the segmentation task, where mask Precision increased from 94.1% to 96.6%, mask *mAP*_50_ increased from 88.9% to 89.8%, and mask *mAP*_50–95_ increased from 63.4% to 64.7%.

Although the Recall of GDFPN was slightly lower than that of the baseline configuration, it remained higher than or comparable to those of the alternative feature fusion networks. More importantly, the consistent improvements in both detection and segmentation accuracy indicate that GDFPN provides more effective multi-scale feature aggregation for chili flower phenotyping. The superior performance under the *mAP*_50_ and *mAP*_50–95_ metrics further suggests that the proposed feature fusion strategy contributes to improved localization accuracy and contour prediction quality in complex field environments.

#### 4.2.4 Benchmark comparisons with state-of-the-art instance segmentation models

To comprehensively evaluate the multi-scale target detection and instance segmentation capabilities of the proposed *Chili-Seg* model in complex field environments, comparative experiments were conducted against several mainstream lightweight instance segmentation frameworks, including YOLOv5n-seg, YOLOv9t-seg, YOLO11n-seg, YOLOv12n-seg, and YOLOv26n-seg.

The quantitative results are summarized in **Table 7**. To provide a more intuitive interpretation of the performance differences, the accuracy–efficiency relationship among the compared models and the progressive accuracy–throughput trade-off during the ablation stages are further visualized in **Figures 9**, **10**, respectively.

**Table 7:** Quantitative performance comparison of the proposed Chili-Seg against mainstream instance segmentation frameworks.

Models	Detect (%)				Mask (%)			
	*P*	*R*	*mAP*_50_	*mAP*_50–95_	*P*	*R*	*mAP*_50_	*mAP*_50–95_
YOLOv5n-seg (Jocher, 2020)	**97.6**	74.9	81	70.3	**97.3**	74.7	80.3	59.1
YOLOv9t-seg (Wang et al., 2024b)	96.9	74.9	79.1	69.9	96.6	74.6	78.3	58.7
YOLO11n-seg (Jocher and Qiu, 2024)	95.1	**80.6**	89.4	76.1	94.5	**80.4**	88.7	63.4
YOLOv12n-seg (Tian et al., 2025)	95.5	74.6	78	68.6	95.3	74	77.2	57.8
YOLOv26n-seg (Jocher et al., 2026)	94.3	74.3	77.8	68.8	94.2	74.2	77.2	57.3
Chili-Seg (Ours)	**96.8**	**80.6**	**90.6**	**77.9**	**96.6**	**80.4**	**89.8**	**64.7**
Models			Params(M)		FLOPs(G)		FPS	
YOLOv5n-seg			2.76		10.3		225.1	
YOLOv9t-seg			3.09		44		83.1	
YOLO11n-seg			2.83		9.6		194.3	
YOLOv12n-seg			2.81		9.6		133.2	
YOLOv26n-seg			2.69		9		149.1	
Chili-Seg (Ours)			3.67		10.3		148.1	

**Figure 9:**
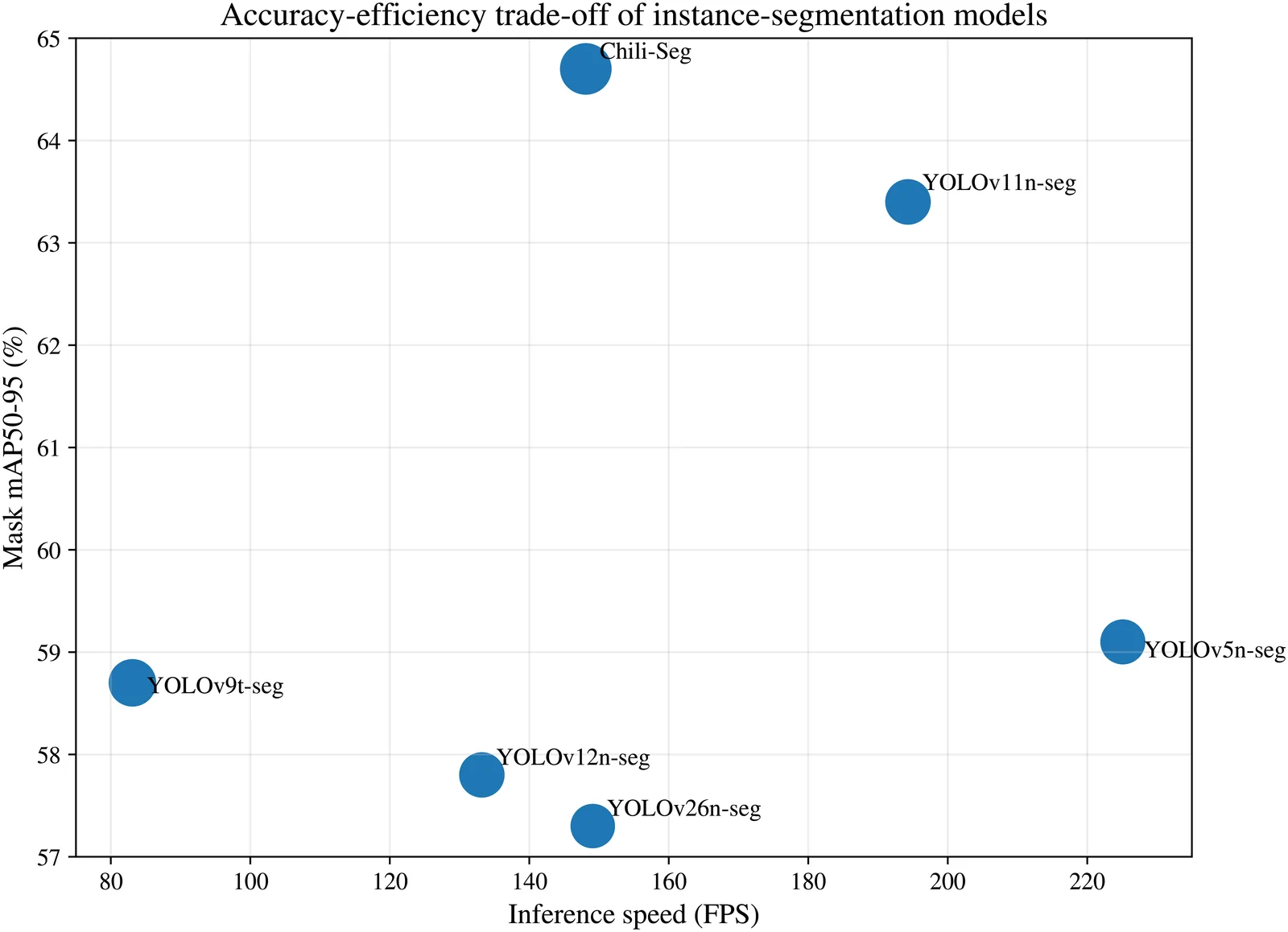
Accuracy–efficiency trade-off among mainstream instance segmentation models. The horizontal axis represents inference speed (FPS), the vertical axis represents Mask *mAP*_50–95_, and the bubble size represents the number of model parameters.

**Figure 10:**
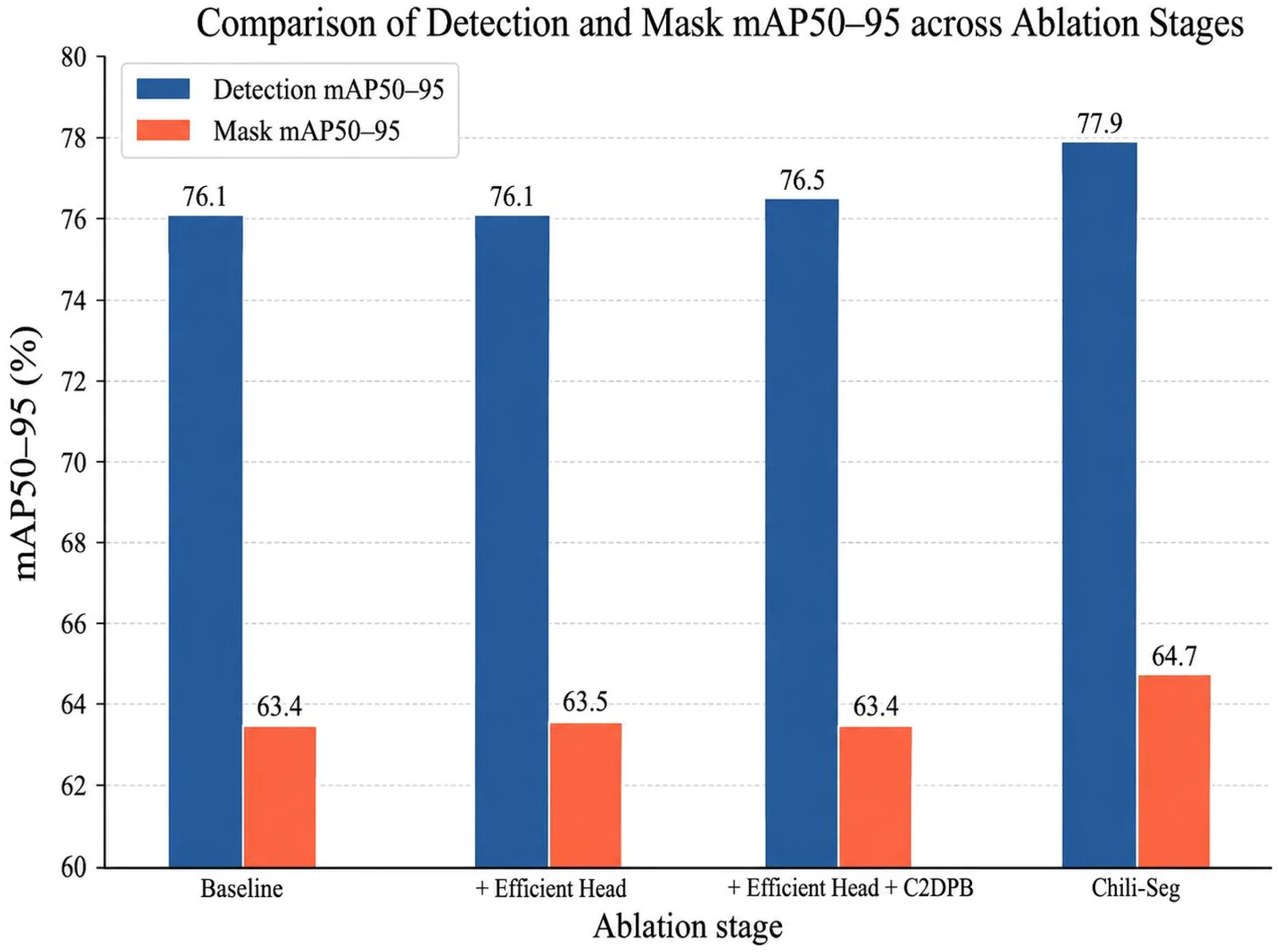
Accuracy–throughput trade-off across the main ablation stages. Box *mAP*_50–95_ and Mask *mAP*_50–95_ are compared for the baseline, +Efficient Head, +Efficient Head+C2DPB, and complete ChiliSeg configurations, with the corresponding FPS reported for each stage.

As shown in **Table 7**, the compared lightweight segmentation models exhibit different trade-offs among Precision, Recall, segmentation accuracy, and computational efficiency. YOLOv5n-seg achieved the highest detection Precision of 97.6% and Mask Precision of 97.3%; however, its Recall remained at approximately 75%, which limited its overall *mAP* performance. Similar tendencies were observed for YOLOv9t-seg, YOLOv12n-seg, and YOLOv26n-seg, whose Recall values also remained below 75%. These results indicate that high Precision alone is insufficient for reliable chili flower perception in complex field environments, where missed detections caused by occlusion, cluttered foliage, and small target size can substantially affect practical performance.

Among the compared baseline architectures, YOLO11n-seg exhibited a more balanced performance. Its detection Recall reached 80.6%, substantially exceeding those of YOLOv5n-seg, YOLOv9tseg, YOLOv12n-seg, and YOLOv26n-seg. Meanwhile, its detection *mAP*_50_ and Mask *mAP*_50_ reached 89.4% and 88.7%, respectively. The relatively favorable balance between target recall and localization/segmentation accuracy therefore provided the basis for selecting YOLO11n-seg as the baseline architecture for subsequent structural optimization.

Based on this baseline, Chili-Seg achieved the best overall *mAP* performance rather than the best value for every individual metric. Although YOLOv5n-seg retained the highest detection and Mask Precision, Chili-Seg achieved the highest detection *mAP*_50_ and *mAP*_50–95_ of 90.6% and 77.9%, respectively, as well as the highest Mask *mAP*_50_ and *mAP*_50–95_ of 89.8% and 64.7%. In addition, its detection and Mask Recall were tied for the highest values among the compared models. These results indicate that Chili-Seg provides a more balanced improvement in target localization and instance contour delineation rather than optimizing Precision alone. In particular, the gains in *mAP*_50–95_ demonstrate improved prediction quality under stricter IoU criteria, reflecting tighter bounding-box localization and better agreement between predicted masks and ground-truth contours.

The accuracy–efficiency distribution in **Figure 9** provides a complementary visualization of the quantitative results in **Table 7**. YOLOv5n-seg achieves the highest inference speed, whereas YOLOv26n-seg has the lowest parameter count and FLOPs; however, both remain in a substantially lower Mask *mAP*_50–95_ region. By comparison, Chili-Seg reaches the highest Mask *mAP*_50–95_ while maintaining an inference speed of 148.1 FPS. The position of Chili-Seg in the accuracy–efficiency space therefore indicates that the improvement in segmentation quality is achieved without reducing the inference throughput to an impractical level, resulting in a favorable balance between prediction accuracy and computational efficiency.

**Figure 10** further illustrates how this trade-off evolves during the progressive integration of the proposed modules. Introducing the Efficient Head initially maintains essentially unchanged Box and Mask *mAP*_50–95_ while increasing the inference speed from 194.3 FPS to 205.8 FPS, indicating that its principal contribution is computational efficiency. After C2DPB is incorporated, detection *mAP*_50–95_ increases while the model retains relatively high throughput. With the subsequent integration of GDFPN, the complete Chili-Seg model achieves the highest Box and Mask *mAP*_50–95_ values, although FPS decreases to 148.1. This progressive trend visually demonstrates that the final performance improvement results from the complementary contributions of the proposed modules and represents an explicit accuracy–efficiency trade-off rather than a gain produced by a single architectural component.

From the perspective of computational cost, YOLOv26n-seg achieved the lowest model complexity, with 2.69 M parameters and 9.0 GFLOPs, while YOLOv5n-seg achieved the highest inference speed of 225.1 FPS. Chili-Seg increased the parameter count to 3.67 M and the computational complexity to 10.3 GFLOPs, while maintaining an inference speed of 148.1 FPS.

Previous real-time vision studies have used 30 FPS as a practical operating reference (Liu and Kang, 2024; Hou et al., 2020). The 148.1 FPS achieved by Chili-Seg remains substantially above this commonly used real-time reference, indicating that the additional computational cost does not compromise its real-time processing capability.

Taken together, **Table 7**; **Figures 9**, **10** provide complementary quantitative and visual evidence of the accuracy–efficiency characteristics of Chili-Seg. Rather than simply repeating individual numerical values, these visualizations make the relative positions of the compared models and the progressive effect of the proposed architectural modifications more intuitive, thereby clarifying the balance between performance gains and computational cost.

#### 4.2.5 Comparative evaluation with mainstream semantic segmentation models

Beyond evaluating instance segmentation networks, this study introduced five classic semantic segmentation algorithms, including U-Net, DeepLabv3+, Pyramid Scene Parsing Network (PSPNet), High-Resolution Network (HRNet), and SegFormer, to conduct a multi-dimensional comparative analysis. This investigation aims to evaluate the distinct impacts of various receptive field aggregation strategies on pixel-level classification precision for chili flower phenotypes.

The quantitative comparison results presented in **Table 8** show noticeable differences in pixel-level segmentation performance among the evaluated semantic segmentation networks. Among all compared methods, PSPNet achieved the lowest performance, with an *mIoU* of 78.83% and an *mPA* of 83.63%.

**Table 8:** Quantitative pixel-level performance comparison among diverse semantic segmentation networks.

Models	*mIoU* (%)	*mPA* (%)
U-Net (Ronneberger et al., 2015)	**92.14**	**94.70**
DeepLabv3+ (Chen et al., 2018)	88.30	91.65
PSPNet (Zhao et al., 2017)	78.83	83.63
HRNet (Wang et al., 2021)	89.03	91.59
SegFormer (Xie et al., 2021)	90.11	92.75

Deeplabv3+ and HRNet obtained intermediate results, reaching *mIoU* values of 88.30% and 89.03%, respectively. Segformer further improved the segmentation accuracy, achieving an *mIoU* of 90.11% and an *mPA* of 92.75%.

Among all evaluated semantic segmentation models, UNet achieved the best overall performance, obtaining an *mIoU* of 92.14% and an *mPA* of 94.70%. Compared with Deeplabv3+, PSPNet, HRNet, and Segformer, the *mIoU* of UNet increased by 3.84, 13.31, 3.11, and 2.03 percentage points, respectively. Similar improvements were observed for the *mPA* metric. These results indicate that UNet provides more accurate pixel-level classification for chili flower segmentation in complex field environments.

Although UNet demonstrated superior semantic segmentation performance, it remains a category-level segmentation method that assigns the same label to all pixels belonging to a given class. Consequently, it cannot distinguish individual flowers when multiple targets overlap or appear in close proximity. In contrast, the proposed Chili-Seg framework performs instance-level segmentation, enabling simultaneous localization and segmentation of individual chili flowers. Therefore, in addition to accurate pixel-level boundary extraction, Chili-Seg can provide independent object identities and spatial information for each flower, which is essential for downstream applications such as automated phenotyping, flower counting, and robotic pollination.

### 4.3 Visual analysis of phenotypic detection and segmentation results

Within unstructured protected agricultural facilities, the visual characteristics of chili flowers undergo severe degradation due to abrupt illumination fluctuations, such as deep shadows or localized overexposure, and complex physical occlusions arising from overlapping branches and foliage. These adverse conditions present severe challenges to the feature representation capabilities and structural robustness of instance segmentation networks. To qualitatively evaluate the generalization efficacy of the proposed *Chili-Seg* framework, its predictive performance within complex field scenarios was visualized and horizontally benchmarked against mainstream baseline models, including YOLOv5n-seg, YOLOv9t-seg, YOLO11nseg, and YOLOv12n-seg, as illustrated in **Figure 11**. In the visualization matrix, red circles highlight the false-positive errors (misdetections and false segmentations) committed by the models, while green circles indicate false-negative errors (missed targets and incomplete segmentations).

**Figure 11:**
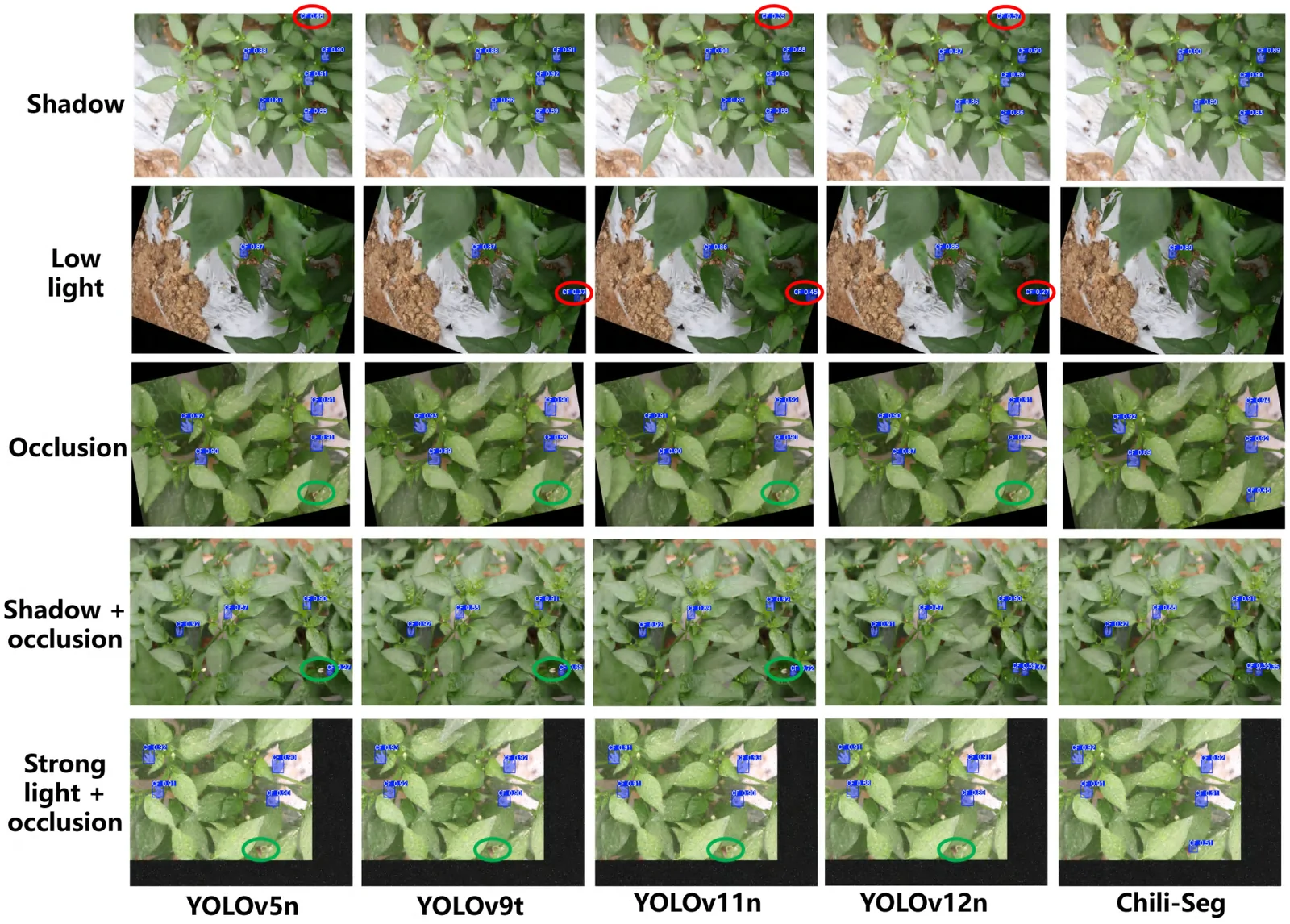
Comparison of the visual detection performance of different models under various complex conditions including shadow, low light and foliage occlusion.

Under shadowed and low-light field conditions, the textual and edge information of minuscule floral buds decays dramatically, and specular reflections from the background soil or smooth leaf surfaces introduce severe visual noise. As shown in the first two rows of **Figure 11**, the alternative YOLO frameworks exhibit extreme sensitivity to illumination degradation. Indicated by the red circles, because these networks fail to effectively differentiate low-contrast targets from ambient background noise, they frequently misclassify specular soil patches or highlighted leaves as floral targets, resulting in high false-positive rates. In sharp contrast, the *Chili-Seg* model executes flawless predictions without committing any false-positive errors under identical conditions, showcasing superior illumination robustness. From an algorithmic standpoint, this success is heavily credited to the C2DPB dual-path feature extraction mechanism integrated into the backbone network. By utilizing a dynamic position bias generator, this module accurately isolates the low-frequency background noise induced by uneven field lighting while fortifying the network’s focus on high-frequency critical traits, such as petal texture profiles, which achieves high-fidelity feature purification within mottled environments.

When chili flowers encounter compounded illumination-occlusion variations, such as shaded-occluded or hyper-illuminated-occluded scenarios, their morphological characteristics are physically truncated, destroying the complete geometric topology of the targets. As shown in the final three rows of **Figure 11**, when flowers are heavily occluded by adjacent foliage, mainstream YOLO models suffer from semantic dispersion of residual features in deep convolutional layers, making them incapable of executing complete instance inferences. As highlighted by the green circles, YOLOv5n-seg, YOLOv9t-seg, YOLO11n-seg, and YOLOv12n-seg consistently fail to detect the severely occluded targets situated in the lower-right regions of the images. Conversely, the *Chili-Seg* architecture delivers outstanding performance when parsing heavily occluded small-scale targets, successfully identifying and segmenting all target instances. This empirical outcome is consistent with the intended role of the GDFPN neck in improving information exchange between shallow high-resolution spatial details and deeper contextual features through dynamic crossscale interactions. This operational mechanism substantially amplifies the network’s contextual reasoning capabilities, allowing it to leverage subtle residual features alongside global topological correlations to accurately extrapolate and reconstruct the complete mask contours of occluded flowers, thereby ensuring sample integrity for high-throughput chili flower phenotyping workflows.

### 4.4 Heatmap interpretability analysis via Grad-CAM

To further elucidate the underlying mechanism driving the performance enhancements of the *Chili-Seg* model from the perspective of network decision explainability, this study utilized the Gradient-weighted Class Activation Mapping (Grad-CAM) technique (Selvaraju et al., 2017). Feature activation heatmaps were extracted to visualize the network’s responsiveness under complex field scenarios, specifically deep shadows and severe foliar occlusions, as illustrated in **Figure 12**. Within this visualization scheme, deep crimson domains represent regions featuring high-dimensional feature activation where the network allocates maximum predictive weight, whereas dark blue zones denote minimal responsiveness.

**Figure 12:**
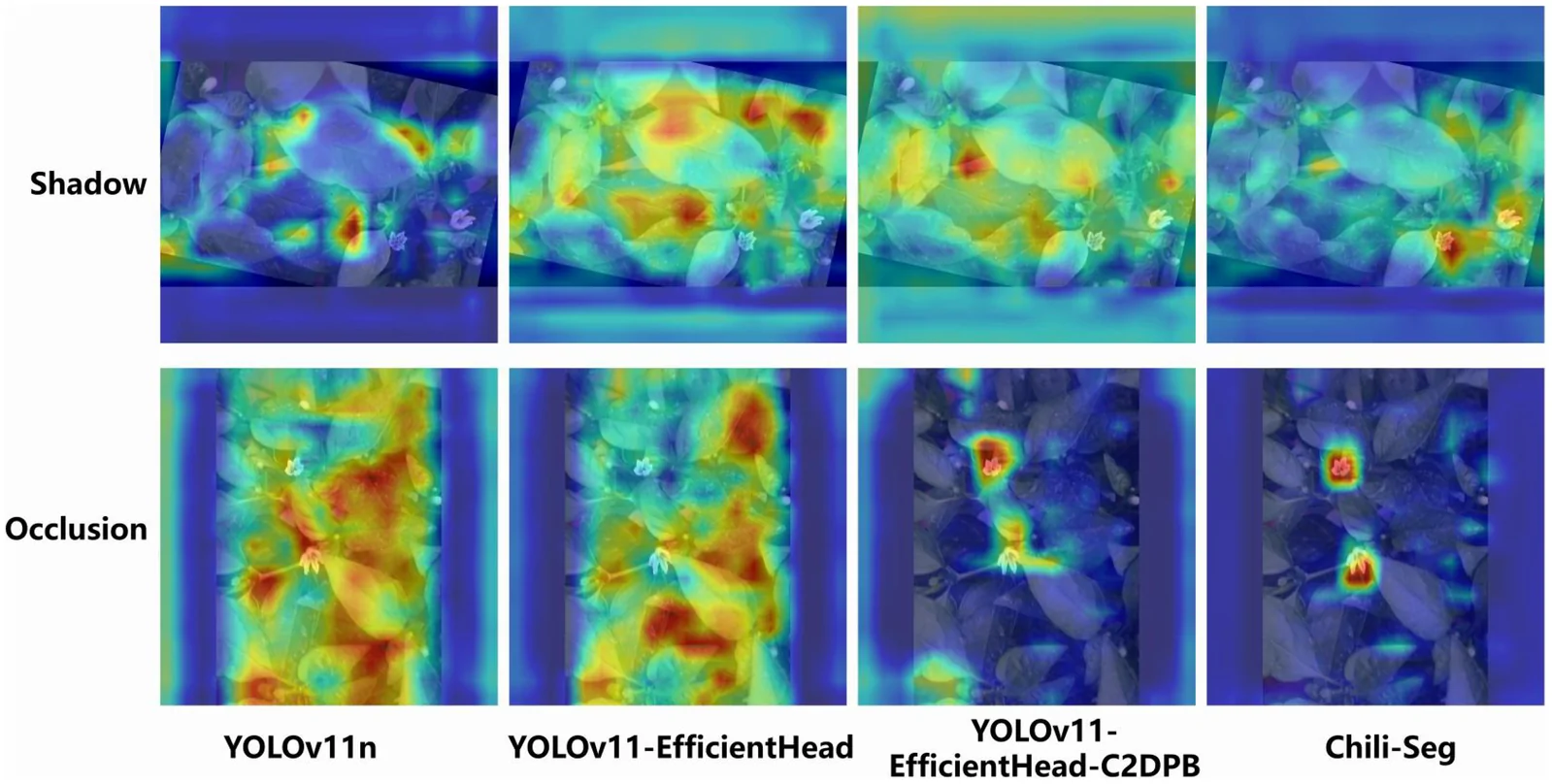
Gradient-weighted Class Activation Mapping (Grad-CAM) visualization results of different improved models under shadow and occlusion scenarios.

A horizontal inspection of the baseline YOLO11n-seg model in the first column clearly reveals a pervasive phenomenon of semantic divergence regarding its attentional distribution. Across both shaded and occluded environments, the primary high-response zones extensively wander and overlay onto the background foliage rather than consolidating their perceptual computation allocation onto the minuscule chili flower bodies. This severe swallowing of target traits by background artifacts provides a fundamental explanation for the elevated false-negative rates exhibited by the baseline model in adverse environments. Upon incorporating only the Efficient Decoupled Head (as depicted in the second column), although the model reorganizes its attentional focus between classification and localization tasks, it still fails to isolate the target effectively against highly similar unstructured backdrops.

After introducing the C2DPB module, the Grad-CAM responses become more concentrated on the chili flower regions, particularly around the corolla margins and central floral structures, while the activation on surrounding foliage is visibly reduced under shaded and occluded conditions. This qualitative observation complements the ablation results, where the combination of the Efficient Head and C2DPB increases the detection Recall from 81.0% to 81.8%, the highest value among the evaluated configurations. Although C2DPB alone does not improve the overall mAP, the combined quantitative and visualization evidence is consistent with enhanced sensitivity to morphology-related features and reduced interference from complex background regions. Therefore, the role of C2DPB is interpreted as a complementary feature-enhancement mechanism rather than an independently dominant source of accuracy improvement.

Ultimately, the fully executed *Chili-Seg* model in the fourth column exhibits ultimate spatial focus and noise-rejection purity. In the deeply challenging low-contrast shadow scenario where all antecedent ablation variants fail to register the target, *Chili-Seg* precisely elicits localized, intense high-response hot spots uniquely within the dark right-hand region of the image, perfectly anchoring the floral body. Under the occlusion scenario, its activated zones adhere tightly to the pistil and stamen centers of the dual flowers, leaving the background almost entirely in a pure blue, low-response state. When Module C (GDFPN) was independently introduced, the detection *mAP*_50_ and *mAP*_50–95_ increased from 89.4% and 76.1% to 90.0% and 76.9%, respectively, while the mask *mAP*_50_ and *mAP*_50–95_ increased from 88.7% and 63.4% to 89.4% and 64.1%. The consistent improvements in both detection and instance segmentation, particularly under stricter IoU thresholds, are consistent with improved multi-scale feature aggregation and better preservation of structural information during feature fusion. From the perspective of Explainable Artificial Intelligence (XAI), these findings rigorously substantiate the superior noise-immunity foundations and high-fidelity instance perception capabilities of the proposed *Chili-Seg* model within extreme agricultural environments.

### 4.5 Deployment experiment

To investigate the deployment feasibility of Chili-Seg in practical agricultural applications, the trained model was deployed on an NVIDIA Jetson AGX Orin edge computing platform and evaluated under realtime operating conditions. 16-bit floating-point (FP16) inference was employed to leverage the hardware acceleration capability provided by the embedded Tensor Cores, thereby improving inference efficiency while maintaining segmentation accuracy. The evaluation scenario was designed to emulate the continuous image acquisition process of an intelligent breeding robot operating in field environments. The deployment interface and runtime information are illustrated in **Figure 13**.

**Figure 13:**
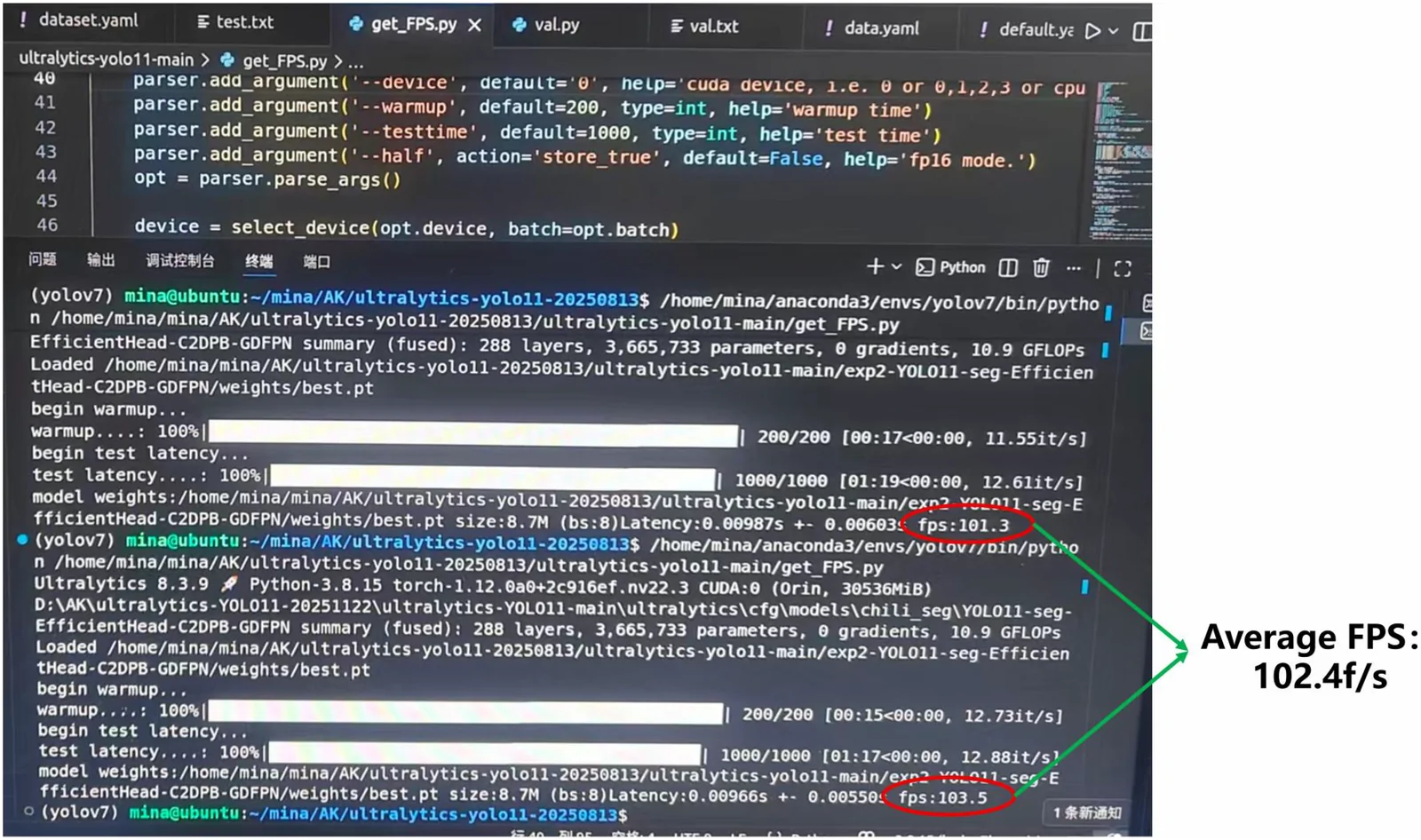
Deployment experiment on NVIDIA Jetson AGX Orin embedded platform.

The experimental results demonstrate that Chili-Seg exhibits remarkable inference efficiency on the edge platform. During continuous operation, the model maintained a stable processing speed between 101.3 and 103.5 FPS, yielding an average throughput of 102.4 FPS. The corresponding average end-to-end latency was 9.7 ms per image. This throughput is well above the 30-FPS operating reference used in prior real-time vision studies (Liu and Kang, 2024; Hou et al., 2020), supporting timely visual perception during dynamic field operations. The results confirm that Chili-Seg can effectively balance segmentation accuracy and computational efficiency on resource-limited hardware. Therefore, the proposed model provides a practical solution for real-time chili flower phenotyping and offers strong potential for deployment in intelligent breeding robots requiring rapid visual guidance and high-throughput phenotypic information acquisition.

### 4.6 Discussion and limitation

The experimental results indicate that the performance improvement of Chili-Seg is not attributable to a single module, but rather to the complementary effects of the three architectural modifications. The Efficient Head primarily improves computational efficiency by reducing parameter redundancy and FLOPs while maintaining essentially unchanged detection and instance segmentation performance. C2DPB provides complementary spatial feature enhancement: although its standalone accuracy gain is limited, its combination with the Efficient Head yields the highest detection Recall among the ablation configurations, while the Grad-CAM visualizations show more concentrated responses on flower regions and reduced activation over surrounding foliage. These observations are consistent with improved sensitivity to morphology-related features under occlusion and complex backgrounds.

Among the three modifications, GDFPN contributes more directly to the improvement of strict-IoU performance. When introduced independently, it produces consistent gains in both detection and mask mAP, and its integration with the Efficient Head and C2DPB further improves detection and mask *mAP*_50–95_. Compared with the gains observed at *mAP*_50_, the more pronounced improvements under stricter IoU thresholds suggest that the fused multi-level features contribute to more accurate localization and better mask overlap. Considering that GDFPN explicitly performs cross-level feature interaction and dynamic resampling, these results are consistent with improved multi-scale feature aggregation and better preservation of discriminative structural information. However, since scale-stratified AP and direct feature-level analyses were not conducted, this interpretation should be regarded as evidence-supported rather than direct proof of the underlying mechanism.

The overall results also reveal an explicit accuracy–efficiency trade-off. The complete Chili-Seg model does not maximize every individual metric; for example, some intermediate configurations achieve higher Recall or inference speed. Nevertheless, Chili-Seg achieves the best overall *mAP*_50_ and *mAP*_50–95_ performance for both detection and masks. This indicates that the final architecture favors more reliable localization and instance delineation rather than optimizing a single metric in isolation. Although the additional feature-fusion operations increase computational cost relative to the Efficient Head configuration, the model still retains a relatively compact parameter scale and high inference throughput, resulting in a balanced compromise between prediction quality and computational efficiency.

The distinction between detection and mask results further clarifies the practical effect of the proposed framework. Box metrics primarily reflect whether chili flowers are correctly detected and spatially localized, whereas mask metrics evaluate pixel-level agreement between predicted instances and ground-truth contours. The consistently higher Box performance indicates that target discovery is generally easier than precise boundary delineation, while the improvements in mask *mAP*_50–95_ demonstrate that Chili-Seg also benefits the more demanding contour-level segmentation task. This distinction is particularly important for chili flower phenotyping, where a correctly detected flower may still provide inaccurate morphological measurements if its instance boundary is poorly segmented.

Despite these improvements, several limitations remain. First, performance degradation is still observed in extremely dense scenes where adjacent flowers are heavily overlapped or physically connected, occasionally leading to ambiguous mask assignments near object boundaries. This suggests that two-dimensional RGB information alone may be insufficient for resolving severe inter-instance occlusion and complex spatial topology. Second, the current dataset was collected from a limited number of chili varieties and planting environments, and cross-variety, cross-region, and cross-device validation has not yet been conducted. Third, the present study does not include scale-stratified AP analysis or direct feature-level measurements, which limits the extent to which the internal mechanisms of the proposed multi-scale feature-fusion strategy can be quantitatively verified.

Future work will therefore focus on extending the current two-dimensional phenotypic perception framework toward three-dimensional and multi-view scene understanding. RGB-D sensing may provide complementary geometric cues for separating closely adjacent flower instances and improving boundary localization under severe occlusion. Flower keypoint estimation and multi-view observations collected during robotic field operations will also be investigated to improve the localization of biologically relevant structures. In addition, the dataset will be expanded to include multiple chili varieties, planting regions, imaging devices, and publicly available agricultural flower datasets, enabling more rigorous cross-domain and cross-dataset evaluation of model generalization. These developments are expected to further improve the robustness of chili flower phenotyping and support downstream applications such as crop monitoring, robotic pollination, and precision breeding.

## 5 Conclusion

This study proposed Chili-Seg, a high-accuracy instance segmentation model for chili flower phenotyping in unstructured field environments, aiming to address the challenges posed by small target size, severe occlusion from branches and leaves, and complex illumination conditions encountered by intelligent breeding robots. By integrating the Efficient Head decoupled prediction architecture, the C2DPB feature extraction module, and the GDFPN multi-scale feature aggregation network, the proposed model significantly enhanced feature representation and segmentation capability for small and densely distributed chili flowers. Experimental results on a self-constructed chili flower dataset demonstrated that ChiliSeg achieved detection precision, mAP_50_, and *mAP*_50–95_ of 96.8%, 90.6%, and 77.9%, respectively, while the corresponding segmentation metrics reached 96.6%, 89.8%, and 64.7%. Compared with the baseline model, the final configuration improved the principal mAP metrics, while five-seed repeated training showed small standard deviations, supporting the reproducibility of the reported performance trend. Furthermore, deployment experiments conducted on the NVIDIA Jetson AGX Orin platform demonstrated excellent computational efficiency, achieving a stable inference speed of 102.4 FPS with low latency, thereby satisfying the real-time requirements of robotic visual perception and field operation tasks. These results indicate that Chili-Seg provides an effective and practical solution for high-throughput chili flower phenotyping and precision breeding applications in complex agricultural environments. Nevertheless, challenges remain in accurately resolving heavily overlapped flowers under dense growth conditions. Future research will therefore focus on integrating RGB-D sensing and keypoint localization techniques to enable three-dimensional phenotypic perception and pollination site localization. In addition, quantitative phenotypic trait extraction, including corolla area, bud expansion degree, flowering index, and other reproductive growth indicators, will be investigated to further support data-driven intelligent breeding and precision cultivation.

## Data Availability

The raw data supporting the conclusions of this article will be made available by the authors, without undue reservation.

## References

[B1] Araus J. L. Cairns J. E. (2014). Field high-throughput phenotyping: The new crop breeding frontier. Trends Plant Sci. 19, 52–61. doi: 10.1016/j.tplants.2013.09.008 24139902

[B2] Bochkovskiy A. Wang C.-Y. Liao H.-Y. M. (2020). Yolov4: Optimal speed and accuracy of object detection. arXiv preprint arXiv:2004.10934. doi: 10.48550/arXiv.2004.10934

[B3] Chen L.-C. Zhu Y. Papandreou G. Schroff F. Adam H. (2018). Encoder-decoder with atrous separable convolution for semantic image segmentation. Comput. Vision – ECCV 2018 11211, 833–851. doi: 10.1007/978-3-030-01234-249

[B4] Cheng B. Misra I. Schwing A. G. Kirillov A. Girdhar R. (2022). “ Masked-attention mask transformer for universal image segmentation”, in: Proceedings of the IEEE/CVF Conference on Computer Vision and Pattern Recognition, 1290–1299. doi: 10.1109/CVPR52688.2022.00135

[B5] Dong Z. Long W. Yang F. Yu C. Du J. Wang K. . (2025). Fruit detection and yield estimation in camellia oleifera based on improved yolov8 and bytetrack algorithm. Smart Agric. Technol. 12, 101435. doi: 10.1016/j.atech.2025.10143542574925

[B6] Dosovitskiy A. Beyer L. Kolesnikov A. Weissenborn D. Zhai X. Unterthiner T. . (2021). “ An image is worth 16x16 words: Transformers for image recognition at scale”, in: International Conference on Learning Representations.

[B7] Fiorani F. Schurr U. (2013). Future scenarios for plant phenotyping. Annu. Rev. Plant Biol. 64, 267–291. doi: 10.1146/annurev-arplant-050312-120137 23451789

[B8] Ge Z. Liu S. Wang F. Li Z. Sun J. (2021). Yolox: Exceeding yolo series in 2021. arXiv preprint arXiv:2107.08430. doi: 10.48550/arXiv.2107.08430

[B9] He K. Gkioxari G. Dollár P. Girshick R. (2017). “ Mask r-cnn”, in: Proceedings of the IEEE International Conference on Computer Vision, 2961–2969. doi: 10.1109/ICCV.2017.322

[B10] Hou R. Li J. Bhargava A. Raventos A. Guizilini V. Fang C. . (2020). “ Real-time panoptic segmentation from dense detections”, in: Proceedings of the IEEE/CVF Conference on Computer Vision and Pattern Recognition, 8523–8532. doi: 10.1109/CVPR42600.2020.00855

[B11] Jain J. Li J. Chiu M. T. Hassani A. Orlov N. Shi H. (2023). “ Oneformer: One transformer to rule universal image segmentation”, in: Proceedings of the IEEE/CVF Conference on Computer Vision and Pattern Recognition, 2989–2998. doi: 10.1109/CVPR52729.2023.00292

[B12] Jiang Y. Li C. (2020). Convolutional neural networks for image-based high-throughput plant phenotyping: A review. Plant Phenomics 2020, 4152816. doi: 10.34133/2020/4152816 33313554 PMC7706326

[B13] Jin X. Yang W. Doonan J. H. Atzberger C. (2022). Crop phenotyping studies with application to crop monitoring. Crop J. 10, 1221–1223. doi: 10.1016/j.cj.2022.09.00142574925

[B14] Jocher G. (2020). Ultralytics yolov5. Software, version 7.0. doi: 10.5281/zenodo.3908559

[B15] Jocher G. Qiu J. (2024). Ultralytics yolo11. Software, version 11.0.0.

[B16] Jocher G. Chaurasia A. Qiu J. (2023). Ultralytics yolov8. Software, version 8.0.0.

[B17] Jocher G. Qiu J. Liu M. Lyu S. Akyon F. C. Kalfaoglu M. E. (2026). Ultralytics yolo26: Unified real-time end-to-end vision models. arXiv preprint arXiv:2606.03748. doi: 10.48550/arXiv.2606.03748

[B18] Kamilaris A. Prenafeta-Boldú F. X. (2018). Deep learning in agriculture: A survey. Comput. Electron. Agric. 147, 70–90. doi: 10.1016/j.compag.2018.02.01642574925

[B19] Kirillov A. Mintun E. Ravi N. Mao H. Rolland C. Gustafson L. . (2023). “ Segment anything”, in: Proceedings of the IEEE/CVF International Conference on Computer Vision, 4015–4026. doi: 10.1109/ICCV51070.2023.00371

[B20] Kuang M. Chen X. Li X. Zou X. Li X. Xiang Y. . (2026a). Pepper disease and pest identification algorithm based on pdpi-yolo. Trans. Chin. Soc. For. Agric. Machinery 57, 262–274. doi: 10.6041/j.issn.1000-1298.2026.10.026

[B21] Kuang M. Li X. Chen S. Liu D. Xiang Y. Liu F. . (2025). Lightweight chili flower target detection method based on improved yolov8n. Trans. Chin. Soc. Agric. Eng. 41, 198–207. doi: 10.11975/j.issn.1002-6819.202502114

[B22] Kuang M. Li X. Wu B. Liu D. Xiang Y. Liu F. . (2026b). Cf-detr: A robust transformer-based framework for small-scale chili flower detection in industrial chili production systems. Front. Plant Sci. 17, 1824412. doi: 10.3389/fpls.2026.1824412 42179510 PMC13190429

[B23] Kuang M. Li X. Xie F. Chen S. Liu D. Xiang Y. . (2026c). Dcfp-yolo: A dual-backbone feature fusion network for multi-pose chili flower recognition and edge deployment. Agriculture 16, 1422. doi: 10.3390/agriculture1613142230654563

[B24] Kuang M. Li X. Xie F. Zou X. Xiang Y. Zhang Y. . (2026d). Advancements and prospects in key technologies for robotic pollination in greenhouse pepper breeding: A review. Front. Plant Sci. 17, 1778541. doi: 10.3389/fpls.2026.1778541 41835286 PMC12982380

[B25] Kuang M. Wang Y. Li X. Liu D. Xiang Y. Liu F. . (2026f). Cfpr-yolo: Chili flower pose estimation for robotic pollination in unstructured environments. Front. Plant Sci. 17, 1861155. doi: 10.3389/fpls.2026.1861155 42328072 PMC13279318

[B26] Kuang M. Wang B. Li X. Zhou J. Liu D. Xiang Y. . (2026e). Cs-obb: An improved oriented bounding box framework for fruit-stem detection and picking point localization of fresh chili peppers. Smart Agric. Technol. 15, 102439. doi: 10.1016/j.atech.2026.10243942574925

[B27] Kuang M. Wang Y. Li X. Zou X. Liu D. Xiang Y. . (2026g). Gcp-yolo: High-precision detection of tiny chili flowers in complex greenhouse scenes. Front. Plant Sci. 17, 1879612. doi: 10.3389/fpls.2026.1879612 42519601 PMC13381778

[B28] Kuang M. Xie F. Liu D. Wu B. Chen S. Xiang Y. . (2026h). A refined yolov5n-based method for detecting pepper flower objects integrating transfer learning. Appl. Soft Comput. 188, 114400. doi: 10.1016/j.asoc.2025.11440042574925

[B29] Kuang M. Xie F. Zou X. Wu B. Liu D. Zou X. . (2026i). Ds-yolo: An improved oriented object detection model for joint localization of mature daylily fruits and stems in unstructured environments. Appl. Soft Comput. 201, 115541. doi: 10.1016/j.asoc.2026.11554142574925

[B30] Kuang M. Zhang Y. Xie F. Liu D. Xiang Y. Li X. . (2026j). Cmd-yolo: An iot-oriented lightweight vision framework for real-time chili maturity detection and robotic localization on edge devices. IEEE Internet Things J. 13, 1–1. doi: 10.1109/jiot.2026.371793225079929

[B31] Kuang M. Zou X. Xie F. Li X. Chen S. Liu D. . (2026k). Ddm-yolo: A lightweight oriented detection model for mature daylily fruits in complex environments. J. King Saud Univ. Comput. Inf. Sci. 38, 91. doi: 10.1007/s44443-026-00559-z30311153

[B32] Li C. Li L. Jiang H. Weng K. Geng Y. Li L. . (2022). Yolov6: A single-stage object detection framework for industrial applications. arXiv preprint arXiv:2209.02976. doi: 10.48550/arXiv.2209.02976

[B33] Li L. Zhang Q. Huang D. (2014). A review of imaging techniques for plant phenotyping. Sensors 14, 20078–20111. doi: 10.3390/s141120078 25347588 PMC4279472

[B34] Liu W. Lu H. Fu H. Cao Z. (2023). Learning to upsample by learning to sample. Proc. IEEE/CVF Int. Conf. Comput. Vision, 6027–6037. doi: 10.1109/ICCV51070.2023.0055425079929

[B35] Liu Y. Kang K.-D. (2024). Filtering empty video frames for efficient real-time object detection. Sensors 24, 3025. doi: 10.3390/s24103025 38793880 PMC11125236

[B36] Liu Z. Lin Y. Cao Y. Hu H. Wei Y. Zhang Z. . (2021). “ Swin transformer: Hierarchical vision transformer using shifted windows”, in: Proceedings of the IEEE/CVF International Conference on Computer Vision, 10012–10022. doi: 10.1109/ICCV48922.2021.00986

[B37] Long J. Shelhamer E. Darrell T. (2015). “ Fully convolutional networks for semantic segmentation”, in: Proceedings of the IEEE Conference on Computer Vision and Pattern Recognition, 3431–3440. doi: 10.1109/CVPR.2015.7298965 27244717

[B38] Lu Y. Zhang Y. Zhang Y. Li D. Bader S. (2026). Pipeline posterior scoring module for out-ofdistribution detection via attachable uncertainty quantification. Reliability Eng. System Saf. 277, 113029. doi: 10.1016/j.ress.2026.113029

[B39] Minervini M. Scharr H. Tsaftaris S. A. (2015). Image analysis: The new bottleneck in plant phenotyping. IEEE Signal Process Mag. 32, 126–131. doi: 10.1109/MSP.2015.240511125079929

[B40] Mirhaji H. Soleymani M. Asakereh A. Abdanan Mehdizadeh S. (2021). Fruit detection and load estimation of an orange orchard using the yolo models through simple approaches in different imaging and illumination conditions. Comput. Electron. Agric. 191, 106533. doi: 10.1016/j.compag.2021.10653342574925

[B41] Mohanty S. P. Hughes D. P. Salathé M. (2016). Using deep learning for image-based plant disease detection. Front. Plant Sci. 7, 1419. doi: 10.3389/fpls.2016.01419 27713752 PMC5032846

[B42] Moreira G. Neves dos Santos F. Cunha M. (2025). Grapevine inflorescence segmentation and flower estimation based on computer vision techniques for early yield assessment. Smart Agric. Technol. 10, 100690. doi: 10.1016/j.atech.2024.10069042574925

[B43] Paulus S. (2019). Measuring crops in 3d: Using geometry for plant phenotyping. Plant Methods 15, 103. doi: 10.1186/s13007-019-0490-0 31497064 PMC6719375

[B44] Redmon J. Farhadi A. (2018). Yolov3: An incremental improvement. arXiv preprint arXiv:1804.02767. doi: 10.48550/arXiv.1804.02767

[B45] Redmon J. Divvala S. Girshick R. Farhadi A. (2016). “ You only look once: Unified, real-time object detection”, in: Proceedings of the IEEE Conference on Computer Vision and Pattern Recognition, 779–788. doi: 10.1109/CVPR.2016.91

[B46] Ronneberger O. Fischer P. Brox T. (2015). “ U-net: Convolutional networks for biomedical image segmentation”, in: Medical Image Computing and Computer-Assisted Intervention – MICCAI 2015, 234–241. doi: 10.1007/978-3-319-24574-428

[B47] Selvaraju R. R. Cogswell M. Das A. Vedantam R. Parikh D. Batra D. (2017). “ Grad-cam: Visual explanations from deep networks via gradient-based localization”, in: Proceedings of the IEEE International Conference on Computer Vision, 618–626. doi: 10.1109/ICCV.2017.74

[B48] Tian Y. Yang G. Wang Z. Li E. Liang Z. (2020). Instance segmentation of apple flowers using the improved mask r-cnn model. Biosyst. Eng. 193, 264–278. doi: 10.1016/j.biosystemseng.2020.03.00842574925

[B49] Tian Y. Ye Q. Doermann D. (2025). Yolov12: Attention-centric real-time object detectors. arXiv. 38, 78433–457. doi: 10.48550/arXiv.2502.12524

[B50] Wang A. Chen H. Liu L. Chen K. Lin Z. Han J. . (2024a). Yolov10: Real-time end-to-end object detection. Adv. Neural Inf. Process. Syst. 37, 107984–108011. doi: 10.52202/079017-3429

[B51] Wang C.-Y. Bochkovskiy A. Liao H.-Y. M. (2023). “ Yolov7: trainable bag-of-freebies sets new state-of-the-art for real-time object detectors”, in: sProceedings of the IEEE/CVF Conference on Computer Vision and Pattern Recognition, 7464–7475. doi: 10.1109/CVPR52729.2023.00721

[B52] Wang C.-Y. Yeh I.-H. Liao H.-Y. M. (2024b). “ Yolov9: learning what you want to learn using programmable gradient information”, in: Computer Vision – ECCV 2024, 1–21. doi: 10.1007/978-3-031-72751-1_1

[B53] Wang D. He D. (2022). Fusion of mask rcnn and attention mechanism for instance segmentation of apples under complex background. Comput. Electron. Agric. 196, 106864. doi: 10.1016/j.compag.2022.10686442574925

[B54] Wang J. Sun K. Cheng T. Jiang B. Deng C. Zhao Y. . (2021). Deep high-resolution representation learning for visual recognition. IEEE Trans. Pattern Anal. Mach. Intell. 43, 3349–3364. doi: 10.1109/TPAMI.2020.2983686 32248092

[B55] Wang W. Yao L. Chen L. Lin B. Cai D. He X. . (2022a). “ Crossformer: a versatile vision transformer hinging on cross-scale attention”, in: International Conference on Learning Representations.

[B56] Wang X. Zubko V. Onychko V. Wu Z. Zhao M. (2022b). Online recognition and yield estimation of tomato in plant factory based on yolov3. Sci. Rep. 12, 8686. doi: 10.1038/s41598-022-12732-1 35606537 PMC9127091

[B57] Xie E. Wang W. Yu Z. Anandkumar A. Alvarez J. M. Luo P. (2021). “ Segformer: simple and efficient design for semantic segmentation with transformers”, in: Advances in Neural Information Processing Systems, 34, 12077–12090.

[B58] Xu X. Jiang Y. Chen W. Huang Y. Zhang Y. Sun X. (2022). Damo-yolo: a report on real-time object detection design. arXiv preprint arXiv:2211.15444. doi: 10.48550/arXiv.2211.15444

[B59] Yang W. Feng H. Zhang X. Zhang J. Doonan J. H. Batchelor W. D. . (2020). Crop phenomics and high-throughput phenotyping: past decades, current challenges, and future perspectives. Mol. Plant 13, 187–214. doi: 10.1016/j.molp.2020.01.008 31981735

[B60] Yu Y. Zhang K. Yang L. Zhang D. (2019). Fruit detection for strawberry harvesting robot in non-structural environment based on mask-rcnn. Comput. Electron. Agric. 163, 104846. doi: 10.1016/j.compag.2019.06.00142574925

[B61] Zhang Y. Lu Y. Li D. Bader S. Zio E. (2026a). “ Dynamic causal graph network for reliable pipeline leak detection,” in Reliability Engineering & System Safety 275, 112795. doi: 10.1016/j.ress.2026.112795

[B62] Zhang Y. Lu Y. Martinez-Rau L. S. Fan Z. Qiu Q. O’Flynn B. . (2026b). Tinyml-enabled iot edge framework with knowledge distillation for weed classification. IEEE Internet Things J. 12, 27453–66. doi: 10.1109/jiot.2026.367950825079929

[B63] Zhang Y. Lu Y. Martinez-Rau L. S. Qiu Q. Bader S. (2026c). Real-time on-device weed identification using a hardware-efficient lightweight cnn. Front. Plant Sci. 17, 1747863. doi: 10.3389/fpls.2026.1747863 41777389 PMC12950715

[B64] Zhang Y. Nürnberg A. Rau L. S. M. Vu Q. N. P. Lu Y. Oelmann B. . (2026d). Tinyml pipeline for efficient crack classification in uav-based structural health inspections. Sci. Rep. 16, 8964. doi: 10.1038/s41598-026-43534-4 41813915 PMC12987979

[B65] Zhao H. Shi J. Qi X. Wang X. Jia J. (2017). “ Pyramid scene parsing network”, in: Proceedings of the IEEE Conference on Computer Vision and Pattern Recognition, 2881–2890. doi: 10.1109/CVPR.2017.660

